# Safety of SGLT2 Inhibitors: A Pharmacovigilance Study from 2013 to 2021 Based on FAERS

**DOI:** 10.3389/fphar.2021.766125

**Published:** 2021-12-20

**Authors:** Xiang Zhou, Xiaofei Ye, Xiaojing Guo, Dongxu Liu, Jinfang Xu, Fangyuan Hu, Yinghong Zhai, Yongqing Gao, Xiao Xu, Ziwei Dong, Jia He

**Affiliations:** ^1^ School of Medicine, Tongji University, Shanghai, China; ^2^ Department of Health Statistics, Second Military Medical University, Shanghai, China

**Keywords:** SGLT2 inhibitor, important medical events, adverse event, FAERS, pharmacovigilance, disproportionality, diabetes

## Abstract

**Background:** Sodium-glucose co-transporter-2 inhibitors (SGLT2is) are widely used in clinical practice for their demonstrated cardiorenal benefits, but multiple adverse events (AEs) have been reported. We aimed to describe the distribution of SGLT2i-related AEs in different systems and identify important medical event (IME) signals for SGLT2i.

**Methods:** Data from the first quarter (Q1) of 2013–2021 Q2 in FAERS were selected to conduct disproportionality analysis. The definition of AEs and IMEs relied on the system organ classes (SOCs) and preferred terms (PTs) by the Medical Dictionary for Regulatory Activities (MedDRA-version 24.0). Two signal indicators, the reported odds ratio (ROR) and information component (IC), were used to estimate the association between SGLT2is and IMEs.

**Results:** A total of 57,818 records related to SGLT2i, with 22,537 SGLT2i-IME pairs. Most SGLT2i-related IMEs occurred in monotherapy (*N* = 21,408, 94.99%). Significant signals emerged at the following SOCs: “metabolism and nutrition disorders” (*N* = 9,103; IC025 = 4.26), “renal and urinary disorders” (3886; 1.20), “infections and infestations” (3457; 0.85). The common strong signals were observed in diabetic ketoacidosis, ketoacidosis, euglycaemic diabetic ketoacidosis and Fournier’s gangrene. Unexpected safety signals such as cellulitis, osteomyelitis, cerebral infarction and nephrolithiasis were detected.

**Conclusion:** Our pharmacovigilance analysis showed that a high frequency was reported for IMEs triggered by SGLT2i monotherapy. Different SGLT2is caused different types and the association strengths of IMEs, while they also shared some specific PTs. Most of the results are generally consistent with previous studies, and more pharmacoepidemiological studies are needed to validate for unexpected AEs. Based on risk-benefit considerations, clinicians should be well informed about important medical events that may be aggravated by SGLT2is.

## Introduction

Sodium-glucose co-transporter-2 inhibitors (SGLT2is) is a relatively new class of oral hypoglycemic agents indicated for adults with type 2 diabetes, which has been proven to be beneficial in reducing glycosylated hemoglobin, lowering blood pressure, losing weight, and improving blood lipids (Taylor et al., 2019; Sridhar et al., 2020). They have also been reported to be superior to placebo treatments in preventing cardiovascular events, improving end-stage renal disease, and reducing mortality (Bonora et al., 2021; Cahn et al., 2021; Ciardullo et al., 2021). In patients with type 2 diabetes who have atherosclerotic cardiovascular disease or established kidney disease or heart failure, SGLT2is is recommended by the American Diabetes Association (ADA) as the priority choice after metformin (American Diabetes, 2021). Currently, the FDA has approved four gliflozin congeners, named dapagliflozin, canagliflozin, empagliflozin, and ertugliflozin. Ipragliflozin, luseogliflozin, and tofogliflozin were launched in Japan. Remogliflozin has only been approved in India (Kuchay et al., 2021).

However, with their widespread use, AEs related to them have been reported (Puckrin et al., 2018; Ueda et al., 2018; Douros et al., 2020; Rampersad et al., 2020; Caparrotta et al., 2021). The common AEs include diabetic ketoacidosis (DKA), acute kidney injury (AKI), pyelonephritis, capacity depletion, genital fungi, and urinary tract infection (Blau et al., 2017; Fadini et al., 2017; Ueda et al., 2018; Caparrotta et al., 2021; Tuttle et al., 2021). They can be explained by the inherent mechanism of action of the drug (SGLT2is lower blood sugar levels by increasing urinary glucose excretion) or by the fact that the applicable users are generally prone to them. Moreover, the FDA issued warnings that canagliflozin is associated with an increased risk for lower-limb amputation and SGLT2is are associated with Fournier’s gangrene (FG). Canagliflozin may indirectly disrupt the homeostasis of calcium and phosphorus, contributing to weight loss, hypotension, loss of bone mineral density, and increased falls (Fadini and Avogaro, 2017; Zhou et al., 2019; Jackson and Moseley, 2020). FG is an extremely rare but life-threatening bacterial infection, which is also known as perineal necrotizing fasciitis. Although diabetes is a risk factor for developing FG, this infection remains rare in patients with it (Bersoff-Matcha et al., 2019). This number only includes reports submitted to the FDA, so there may be other cases that we are not aware of.

SGLT2is may trigger serious AEs, which may remain undetected. Therefore, a comprehensive analysis of important medical events (IMEs) is necessary to uncover the signals of all SGLT2is-IMEs pairs. This study aimed to analyze IMEs associated with SGLT2is in all SOCs by mining the large pharmacovigilance database (FAERS). We presented more descriptive information on patient characterizations as Supplementary Data to previous studies and mapped signal profiles to visually represent the correlation between SGLT2is and IMEs, to more quickly identify potential safety issues and provide recommendations for clinical use.

## Materials and Methods

### Data Sources and Procedures

The FAERS database is a spontaneous reporting system (SRS) collecting global reports of AEs to support the FDA’s post-marketing surveillance for drugs and biotherapy products. It enables signal detection and quantification of the association between drugs and reporter AEs (Min et al., 2018; Zhai et al., 2019). Data is aggregated quarterly and contains AE reports, medication error reports, and product quality complaints. In this study, we extracted all reports of SGLT2i from FAERS between 2013Q1 (first FDA approval) and 2021Q2 (the latest). Considering the existence of duplicate records in FAERS, we performed the deduplication process. According to the FDA recommendation (Hu et al., 2020), duplicate records are removed by selecting the latest FDA_DT when the CASEID and FDA_DT are the same.

Canagliflozin, empagliflozin, dapagliflozin, ertugliflozin, ipragliflozin, tofogliflozin, luseogliflozin, remogliflozin, and their combination with other drugs all included. Monotherapy here was defined as a specific SGLT2i used alone, which means that this specific SGLT2i is the “primary suspect” in the ROLE_COD field of the DRUG file and without other antidiabetic drugs in the same report as “secondary suspect,” “concomitant” or “interacting.” Combination therapy/polytherapy was defined as concurrent administration of SGLT2is and other antidiabetic agents, which implies that in the same report if the specific SGLT2i is the “primary suspect,” the other antidiabetic agent is the “secondary suspect,” “concomitant” or “interacting,” and vice versa. Since FAERS does not standardize drug names, both brand names and generic names were used to identify records of target drugs. The index of all drug names was shown in Supplementary Table S1. All AEs were coded as preferred terms (PTs) according to MedDRA (*version 24.0*). The primary system organ classes (SOCs) corresponding to these PTs were also listed, and SOCs were equivalent to the system classification in other medical terms. Subsequently, we used the Important Medical Events (IMEs) list to filter out those associated with SGLT2is and mapped a signal profile of SGLT2is-induced IMEs. The list of IMEs is developed by European Medicines Agency (EMA) to help prioritize the review reports of suspected adverse drug reactions (ADRs) in the framework of routine pharmacovigilance activities and is used to identify reports of suspected ADRs which deserve attention. The full IME list is openly available on the website of https://www.ema.europa.eu/documents/other/important-medical-event-terms-list-version-meddra-version-240_en.xlsx. Severe outcomes included life-threatening events or those causing hospitalization, disability, or death. Unexpected AE was defined as any significant adverse event that was not mentioned in the FDA drug prescribing information.

### Statistical Analysis

Disproportionality analysis was performed in our study to indicate the presence of signals of potential increased risk of drug-related AE. In quantitative signal detection, a disproportionately high frequency of drug-event pairs in the database compared to expected may represent a significant signal (van Puijenbroek et al., 2002; Montastruc et al., 2011). We used two disproportional signal detection methods based on the frequency and Bayesian theories - the proportional report odds ratio (ROR) and Bayesian confidence propagation neural network of information components (IC) - to verify the stability of the detected signals. Considering that a spurious association can occur when events with very low expected frequency, statistical shrinkage transformation was performed to obtain conservative results (Noren et al., 2013; Zhai et al., 2019). The shrinkage transformation of ROR and IC is calculated as follows:

$$\text{ROR}=\frac{N_{observed}+0.5}{N_{expected}+0.5}\;\;\;\;\;\;\;\;\;\;$$

$$\text{IC}=\log_{2}\frac{N_{observed}+0.5}{N_{expected}+0.5}\;\;\;$$

$$N_{expected}=\frac{N_{drug}*N_{event}}{N_{total}}$$

$${\text{ROR}}_{025}=e^{\ln\left(ROR\right)-1.96\times \sqrt{\frac{1}{a}+\frac{1}{b}+\frac{1}{c}+\frac{1}{d}}}$$

$${\text{ IC}}_{025}=\text{IC}-3.3\times {\left(N_{observed}+0.5\right)}^{-0.5}-2\times {\left(N_{observed}+0.5\right)}^{-1.5}$$

All the measures of disproportionality are based on the same principles of calculation using the 2 × 2 table (Supplementary Table S2). N_observed_ = a, which is is the observed number of records of interested drug-AE pairs, N_expected_ is the expected number of records of interested drug-AE pairs, N_drug_ is the total number of records of the target drug, N_event_ is the total number of records of target AEs, and N_total_ is the total number of records in the whole database.

The 95% confidence intervals for ROR (ROR_025_) and IC (IC_025_) were calculated to describe the association strength of SGLT2is and IMEs. A significant signal was defined as ROR_025_ greater than 1 with at least three cases or IC_025_ above zero (van Puijenbroek et al., 2002). All analyses were performed SAS version 9.4 (SAS Institute Inc., Cary, NC, United States).

## Results

### Descriptive Analysis

From 2013q1 to 2021q2, a total of 11,822,884 AE reports were submitted to the FDA, with 57,818 related to the use of SGLT2i, of which 22,537 (38.98% to all SGLT2i) reported events were IMEs. The distribution of gliflozin congeners and the clinical characteristics of the patients are described in Tables 1,2. In general, the distribution of each indicator was similar in SGLT2i-related AEs and IMEs. For SGLT2i-related IMEs, monotherapy (94.99%) accounted for a much larger proportion than combination therapy, with canagliflozin constituting nearly half (48.26%). The vast majority of reports cited SGLT2i as the primary suspect drug (94.55%). Most reported cases were male (48.60%). The median age was 58 (interquartile range [IQR] 49–67, and nearly half (47.53%) of the cases were under 65 years old. The median weight of the available data was 84 kg (IQR 70–100 kg). There is an increasing trend overall in reporting years, with peaks in 2015 and 2018. This may be due to the concern raised by the FDA issuing some black box warnings about SGLT2is at these times. Hospitalization and other serious medical events were the most frequently reported outcomes. During the study period (nearly 9 years), 2,464 patients developed severe outcome events (except hospitalization) reported in IME. It implied that SGLT2is may have potentially dangerous attributes.

**TABLE 1:** Distribution of SGLT2i across therapies^a^.

	SGLT2i-related AEs *n* (%)	SGLT2i-related IMEs *n* (%)
Total	57,818	22,537
Monotherapy	54,227 (93.79)	21,408 (94.99)
canagliflozin	25,120 (46.32)	10,331 (48.26)
empagliflozin	16,284 (30.03)	6,083 (28.41)
dapagliflozin	12,292 (22.67)	4,892 (22.85)
ertugliflozin	512 (0.94)	93 (0.43)
ipragliflozin	2 (0.00)	2 (0.01)
tofogliflozin	12 (0.02)	5 (0.02)
sotagliflozin	5 (0.01)	2 (0.01)
Polytherapy	3,591 (6.21)	1,129 (5.01)
Polytherapy1	2,385 (4.13)	812 (3.6)
canagliflozin+metformin	1,064 (44.61)	287 (35.34)
empagliflozin+metformin	1,240 (51.99)	498 (61.33)
dapagliflozin+metformin	37 (1.55)	19 (2.34)
ertugliflozin+metformin	44 (1.84)	8 (0.99)
Polytherapy2	1,184 (2.05)	317 (1.41)
empagliflozin+linagliptin	1,133 (95.69)	300 (94.64)
ertugliflozin+sitagliptin	51 (4.31)	17 (5.36)
Polytherapy3	22 (0.04)	0 (0.00)
empagliflozin+linagliptin+metformin	22 (100.00)	0 (0.00)

a In Table 1, AEs, adverse effects; IMEs, important medical events.

**TABLE 2:** Characteristics of patients with SGLT2i-related AEs/IMEs^a^.

	SGLT2i-related AEs *n* (%)	SGLT2i-related IMEs *n* (%)
Total	57,818	22,537
Gender		
Male	26,375 (45.62)	10,954 (48.60)
Female	25,122 (43.45)	8,701 (38.61)
Unknown or missing	6,321 (10.93)	2,882 (12.79)
Age (year)		
median (IQR)	60 (51–68)	58 (49–67)
<18	73 (0.13)	20 (0.09)
18–64	22,264 (38.51)	10,692 (47.44)
65–84	10,512 (18.18)	4,420 (19.61)
≥85	438 (0.76)	207 (0.92)
Unknown or missing	24,531 (42.43)	7,198 (31.94)
Weight (kg)		
median (IQR)	86.85 (72.60–103.90)	84 (70–100.25)
≤73	3,730 (6.45)	1787 (7.93)
≤87	3,573 (6.18)	1,394 (6.19)
≤104	3,609 (6.24)	1,326 (5.88)
>104	3,531 (6.11)	1,236 (5.48)
Unknown or missing	43,375 (75.02)	16,794 (74.52)
Year		
2013	220 (0.38)	80 (0.35)
2014	2010 (3.48)	416 (1.85)
2015	10,352 (17.90)	1975 (8.76)
2016	7,402 (12.80)	2,873 (12.75)
2017	8,432 (14.58)	4,439 (19.70)
2018	10,394 (17.98)	4,884 (21.67)
2019	8,893 (15.38)	3,672 (16.29)
2020	6,777 (11.72)	2,823 (12.53)
2021 (Q1-Q2)	3,338 (5.77)	1,375 (6.10)
Reporter Country		
United States	41,130 (71.14)	12,865 (57.08)
Japan	3,398 (5.88)	2,059 (9.14)
Great Britain	2,209 (3.82)	1,363 (6.05)
Other countries	11,081 (19.17)	6,250 (27.73)
Outcome		
Death	1,117 (1.93)	935 (4.15)
Life-threatening	1,636 (2.83)	1,167 (5.18)
Hospitalization	16,806 (29.07)	10,877 (48.26)
Disability	995 (1.72)	351 (1.56)
Congenital anomaly	17 (0.03)	11 (0.05)
Other serious	15,629 (27.03)	8,479 (37.62)
Required intervention	42 (0.07)	15 (0.07)
Unknown or missing	21,576 (37.32)	702 (3.11)
Indications		
Type 2 diabetes mellitus	26,013 (44.99)	10,717 (47.55)
Non-specifed diabetes mellitus	9,304 (16.09)	3,928 (17.43)
Type 1 diabetes mellitus	1,050 (1.82)	703 (3.12)
Cardiac disorder	394 (0.68)	201 (0.89)
Other indications	441 (0.76)	137 (0.61)
Unknown or missing	20,616 (35.66)	6,851 (30.4)
Role of drugs		
Primary Suspect	54,789 (94.76)	21,308 (94.55)
Secondary Suspect	3,020 (5.22)	1,226 (5.44)
Concomitant	9 (0.02)	3 (0.01)

a In Table 2, AEs, adverse effects; IMEs, important medical events.

### SGLT2i-Related AEs/IMEs Differ in the Strength of Association With Various SOCs

Considering SGLT2i as a class, the signal strengths of its associated AEs or IMEs at different SOCs are presented in Table 3. Significant signal overlap emerged in three SOCs: “infections and infestations” (IC_025_ of AEs = 1.67; IC_025_ of IMEs = 0.85), “metabolism and nutrition disorders” (3.33; 4.26), “renal and urinary disorders” (1.94; 1.20). In addition, SGLT2i-induced AEs also showed significance in “investigations,” “reproductive system and breast disorders” and “surgical and medical procedures.” Among them, “Reproductive system and breast disorders” had the lowest number of reports but relatively strong association (*N* = 911, IC_025_ = 0.88).

**TABLE 3:** Signal strength of SGLT2i and AEs/IMEs at the System Organ Class (SOC) level^a^.

SOC	SGLT2i-related AEs			SGLT2i-related IMEs		
	*N*	IC_025_	ROR_025_	*N*	IC_025_	ROR_025_
Blood and lymphatic system disorders	186	−2.87	0.14	40	−4.75	0.04
Cardiac disorders	1,631	−0.31	0.81	1,302	−0.85	0.56
Congenital familial and genetic disorders	61	−2.04	0.25	21	−3.16	0.12
Ear and labyrinth disorders	83	−1.56	0.35	28	−2.33	0.21
Endocrine disorders	34	−2.59	0.18	25	−3.22	0.12
Eye disorders	431	−1.32	0.41	182	−1.88	0.28
Gastrointestinal disorders	3,812	−0.49	0.72	659	−1.16	0.45
General disorders and administration site conditions	4,230	−1.54	0.34	549	−2.64	0.16
Hepatobiliary disorders	327	−0.86	0.56	195	−1.69	0.32
Immune system disorders	287	−2.20	0.22	64	−3.21	0.11
Infections and infestations	9,081	**1.67**	**3.19**	3,457	**0.85**	**1.81**
Injury poisoning and procedural complications	1910	−1.59	0.33	65	−2.70	0.16
Investigations	6,483	**0.97**	**1.97**	6	−5.33	0.03
Metabolism and nutrition disorders	11,578	**3.33**	**10.04**	9,103	**4.26**	**19.12**
Musculoskeletal and connective tissue disorders	1,281	−1.09	0.48	56	−3.10	0.12
Neoplasms benign malignant and unspecified	871	−1.36	0.39	775	−1.97	0.26
Nervous system disorders	3,116	−0.43	0.75	1,421	−0.76	0.60
Pregnancy puerperium and perinatal conditions	13	−5.18	0.03	12	−5.31	0.03
Product issues	64	−4.23	0.06	0	-	-
Psychiatric disorders	678	−2.24	0.21	82	−4.18	0.06
Renal and urinary disorders	5,463	**1.94**	**3.85**	3,886	**1.20**	**2.31**
Reproductive system and breast disorders	911	**0.88**	**1.87**	64	−0.44	0.77
Respiratory thoracic and mediastinal disorders	634	−1.92	0.27	133	−2.77	0.15
Skin and subcutaneous tissue disorders	2,520	−0.37	0.78	178	−1.46	0.37
Social circumstances	86	−1.32	0.42	23	−1.64	0.35
Surgical and medical procedures	1,413	**0.58**	**1.51**	0	-	-
Vascular disorders	634	−0.78	0.59	211	−1.78	0.30

a In Table 3, the red bold text denotes significant signals. AEs, adverse effects; IMEs, Important Medical Events; IC025, the lower end of the 95% confidence interval of IC; IC975, the upper end of the 95% confidence interval of IC; ROR025, the lower end of the 95% confidence interval of ROR; ROR975, the upper end of the 95% confidence interval of ROR; IC025 greater than 0 was deemed a signal, ROR025 over 1 with at least three cases was a signal.

### The Spectrum of IMEs Differs in SGLT2i Regimens

**FIGURE 1:**
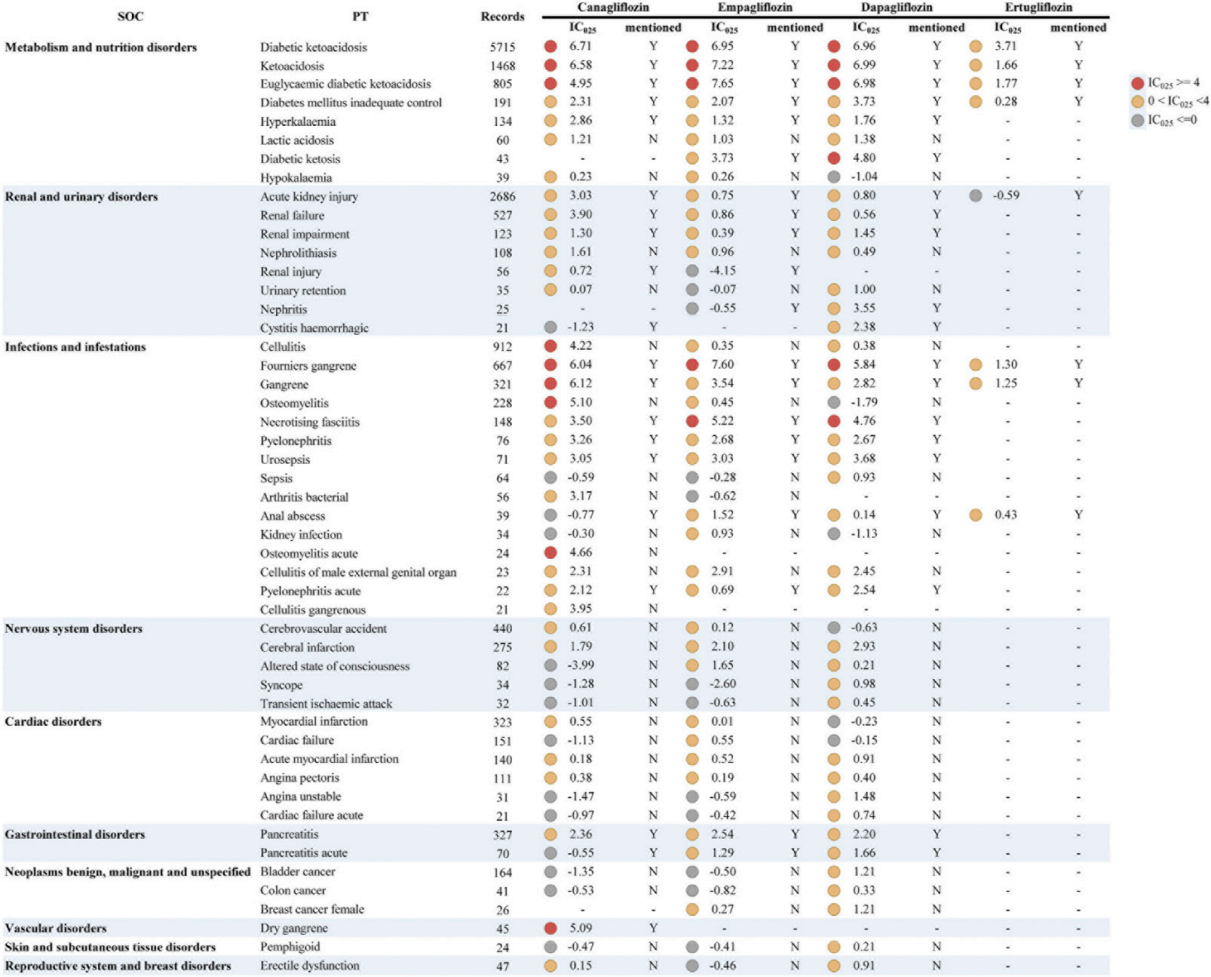
Signal profiles of top 50 IMEs induced by SGLT2i monotherapy-based on all other drugs as “non-case”* *In Figure 1, IMEs, Important Medical Events; SOC, System Organ Class; PT, Preferred Term; IC, information component; IC_025_, the lower limit of the 95% confidence interval of IC. IC_025_ greater than 0 was deemed a signal. IC_025_ no less than 4 was deemed a strong signal.

We further analyzed the different drugs in the monotherapy with each specific IME to explore whether there was an association between them. Since the number of drug-specific IME combinations for ipragliflozin, tofogliflozin, and sotagliflozin was less than 3, these three drugs were not included in this analysis. The top 50 (116 in total) significant signals ranked by frequency are listed in Figure 1. We used the IC_025_ value as an indicator and defined a strong signal when the IC_025_ value was not lower than 4. A full list of IMEs for SGLT2i can be accessed in the Supplementary Figure S1 and Supplementary Table S3. The distribution of significant signals in Figure 1 and Supplementary Figure S1 is approximately the same. The PTs in them were sorted in descending order within the corresponding SOCs.

For the complete signal spectrum, SGLT2i-involved IMEs were mainly distributed in “infections and infestations” (*n* = 40), accordingly, the most signals appeared. Although relatively few IMEs were monitored in “metabolism and nutrition disorders,” the strong signal distribution was the most intensive. This result was consistent with the strength of the association between IMEs and specific SOCs in Table 3. From the specific drug perspective, dapagliflozin presented the broadest spectrum with a total of 69 potential signals detected, ranging from ventricular tachycardia (IC_025_ = 0.06) to ketoacidosis (IC_025_ = 6.99). In addition to the currently known AEs (diabetic ketoacidosis, Fournier’s gangrene, etc.), there remained 46 IMEs not previously mentioned in the prescribing information for dapagliflozin, of which stronger signals were found for epididymitis, cerebral infarction, and brain stem infarction. For canagliflozin, a total of 64 significant signals were observed, with IC_025_ values ranging from 0.02 (bladder transitional cell carcinoma) to 6.71 (diabetic ketoacidosis). Strong signals emerged in ketoacidosis (unspecified type), gangrene (unspecified type), osteomyelitis, and cellulitis. The latter two were not observed in the instructions, and acute osteomyelitis and dry gangrene were unique strong signals for canagliflozin. There were 61 PTs significantly associated with empagliflozin. Of these, euglycaemic diabetic ketoacidosis (euDKA), Fournier’s gangrene (FG), ketoacidosis, diabetic ketoacidosis (DKA), necrotising fasciitis showed a robust correlation (corresponding IC_025_ = 7.65, 7.60, 7.22, 6.95, 5.22) and the first four PTs are shared with canagliflozin and dapagliflozin. However, the drug with the fewest PTs was ertugliflozin, with only eight signals detected and all overlapping with other drugs. Notably, DKA, ketoacidosis, euDKA, FG, gangrene, and “diabetes mellitus inadequate control” were the six PTs common to these four SGLT2is, the first four of which showed reported with high frequency and marked strong intensity.

**FIGURE 2:**
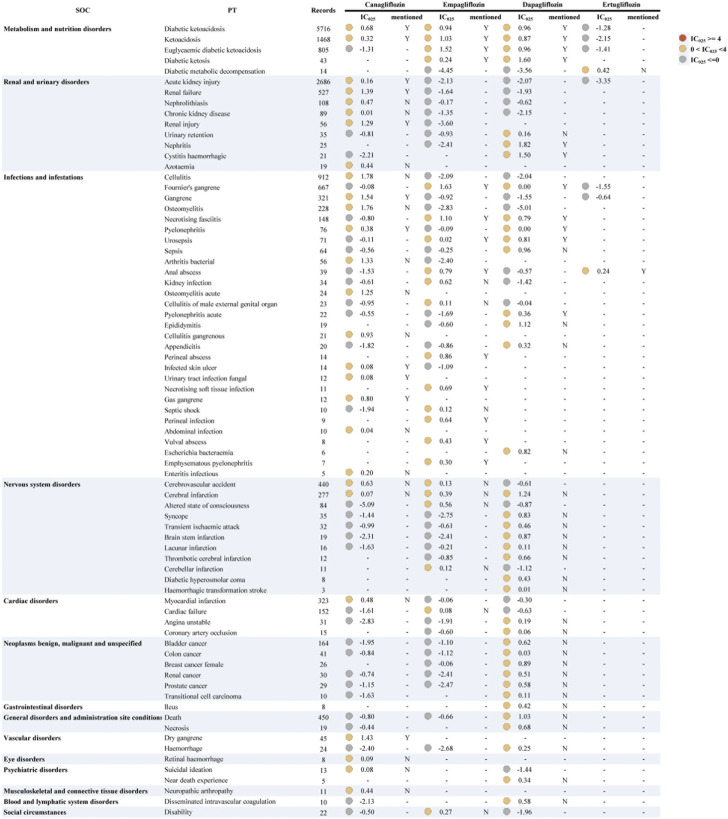
Signal profiles of all IMEs induced by SGLT2i monotherapy-based on other antidiabetic drugs as “non-case”* *In Figure 2, IMEs, Important Medical Events; SOC, System Organ Class; PT, Preferred Term; IC, information component; IC_025_, the lower limit of the 95% confidence interval of IC. IC_025_ greater than 0 was deemed a signal. IC_025_ no less than 4 was deemed a strong signal.

We also used other antidiabetic drugs as “non-cases” to adjust for the background risk of such events in diabetic patients, taking into account the effect of the underlying disease (Figure 2 and Supplementary Table S4). The results indicated a general decrease in IC_025_ values and no strong signal. Similar to all drugs as non-cases, the signals were mostly observed in “infections and infestations.” Likewise, dapagliflozin also had the widest signal spectrum (*n* = 38), ranging from pyelonephritis (IC_025_ = 0.0005) to nephritis (1.82). The strong signal with dapagliflozin found in Figure 1 also appears in Figure 2, but the signal strength is substantially reduced. Nephritis, diabetic ketosis, cystitis haemorrhagic, and cerebral infarction with relatively strong signals (corresponding IC_025_ = 1.82, 1.60, 1.50, 1.24). This was followed by canagliflozin (*n* = 27), where cellulitis, osteomyelitis, and gangrene had the top three signal strengths (1.78, 1.76, 1.54). Notably, the signal of Fournier’s gangrene and euglycaemic diabetic ketoacidosis vanished in canagliflozin and ertugliflozin, while stronger signals were consistently shown in empagliflozin. Cellulitis, osteomyelitis, cerebral infarction, and nephrolithiasis, showed unexpected signals in both analyses.

We presented common significant signals for serious outcomes in monotherapy (Table 4). DKA, ketoacidosis, euDKA, FG, and gangrene were frequently reported. Further analysis indicated that DKA and ketoacidosis seemed to be more strongly associated with canagliflozin, empagliflozin, and dapagliflozin (Supplementary Figure S2). Slightly lower frequency and association of canagliflozin with euDKA compared to empagliflozin and dapagliflozin. A similar scenario occurs for the combination of dapagliflozin with FG. Age below 65 years or men seems to be more likely to suffer from FG (Supplementary Figure S3). Notably, empagliflozin alone or combined metformin\linagliptin was highly associated with developing these four SGLT2i-related IMEs.

**TABLE 4:** Common significant signals for serious outcomes in monotherapy.

Serious adverse event outcome	Common significant signals	Records
Death	Diabetic ketoacidosis	47
	Ketoacidosis	16
	Fournier’s gangrene	13
	Euglycaemic diabetic ketoacidosis	3
	Diabetes mellitus inadequate control	2
Life-threatening	Diabetic ketoacidosis	417
	Ketoacidosis	115
	Euglycaemic diabetic ketoacidosis	100
	Fournier’s gangrene	50
	Gangrene	3
	Diabetes mellitus inadequate control	1
Hospitalization	Diabetic ketoacidosis	3,338
	Ketoacidosis	529
	Euglycaemic diabetic ketoacidosis	410
	Fournier’s gangrene	353
	Gangrene	184
	Diabetes mellitus inadequate control	46
Disability	Gangrene	48
	Fournier’s gangrene	15
	Diabetic ketoacidosis	8
	Diabetes mellitus inadequate control	3
	Ketoacidosis	1
Congenital anomaly	Fournier’s gangrene	1

## Discussion

In this study, the potential adverse signals of SGLT2i were mined based on the FAERS database using ROR and IC methods. The two methods are based on different statistical ideas (ROR belongs to the frequency method and IC is based on the Bayesian method) which can reflect the target drug-AE association in a mutually validated, rapid, and quantitative way. In addition to analyzing all SGLT2i-induced AEs like previous studies (Raschi et al., 2017; Ueda et al., 2018; Caparrotta et al., 2021), we focused on the IMEs associated with SGLT2i, mapping an important potential signal spectrum for monotherapy in a visual manner. Our findings are as follows.

### IMEs That Overlap in Monotherapy and Not Mentioned in the Instructions

According to our analysis, DKA, ketoacidosis, euDKA, FG, gangrene, and “inadequate diabetic control” were the six common IMEs after receiving the four FDA-approved SGLT2is. Further analysis demonstrated that the first five AEs were more likely to occur with SGLT2i compared to other hypoglycemic agents. They were all illustrated in the instructions, and the first four PTs appeared as highly significant signals in all four drugs. These AEs have been proven in previous studies or clinical trials (Ueda et al., 2018; Scheen, 2019; Toyama et al., 2019; Hu et al., 2020). EuDKA is a rare complication of diabetes, with normal or slightly increased blood glucose levels. This often masks the symptoms of DKA and easily leads to a missed diagnosis. The FDA states that regardless of current blood glucose levels (even if blood glucose levels are below 250 mg/dl), physicians should discontinue the drug and handle it as soon as the patient develops signs and symptoms of ketoacidosis. In post-marketing surveillance of diabetic patients receiving SGLT2 inhibitors (Bersoff-Matcha et al., 2019; Fadini et al., 2019; Hu et al., 2020), reports of necrotizing fasciitis of the perineum (Fournier’s gangrene), a rare but serious and life-threatening necrotizing infection requiring urgent surgical intervention, were identified. Cases have been reported in both women and men, with men being more susceptible (Kuchinka et al., 2019; Wang et al., 2020), and we found the same results. In addition, we found a strong association between empagliflozin and FG occurrence whether based on all other drugs or other antiglycemic agents. The same results emerged in a study based on FAERS exploring the association of FG with SGLT2i (Hu et al., 2020).

A total of 11 PTs exist that are not mentioned in the SGLT2i instructions, mainly related to cardio-cerebral diseases and infectious diseases (Cellulitis). For example, “Acute myocardial infarction,” “Angina pectoris,” “Coronary artery stenosis,” and “Cerebral infarction” were identified as significant signals. Notably, compared to other antiglycemic medications, the frequency of cellulitis, osteomyelitis, cerebral infarction, and nephrolithiasis was higher in SGLT2is users. Although SGLT2i has been confirmed to have cardiovascular protective effects, a few studies noted that some patients developed some of these AEs shortly after receiving treatment, and most of these cases occurred in Japan. (Ito et al., 2016; Goda et al., 2018). In contrast, a meta-analysis provided evidence that SGLT2is had no significant effect on ischemic events caused by atherosclerotic coronary artery disease in patients with type 2 diabetes (Ye et al., 2021). Whether SGLT2is contribute to these AEs is controversial and without evidence from large pharmacoepidemiological studies or clinical trials. However, what we do know is that SGLT2i treatment can lead to volume depletion or even dehydration due to excessive diuresis. And dehydration may lead to thromboembolism (e.g., cerebral infarction). Therefore, patients should be encouraged to drink appropriate amounts of water regularly. Meanwhile, cerebral infarction and myocardial infarction are also rare and serious complications of DKA (Yaroglu Kazanci et al., 2015).

### Distribution Characteristics of SGLT2i-AE/IME at the SOC Level

In both SGLT2i-induced AEs and IMEs, we obtained signals of infectious, metabolic, and urinary/renal events, which was generally consistent with the safety data obtained from RCTs (Scheen, 2019; American Diabetes, 2021). It also demonstrated the credible predictive ability of SRS in pharmacovigilance. Thus, before initiating therapy, prescribers should be aware of patients with concomitant diseases of the above systems and keep them under surveillance to avoid deterioration. In addition, an SRS-based study (Raschi et al., 2017) monitored the significance of “skin and subcutaneous tissue disorders,” which was not highlighted in our results.

### Limitations and Strengths

Our study has several limitations. Firstly, FAERS has inherent flaws as an SRS, such as underreporting, duplicate records, uneven information quality, lack of controls, and inability to calculate incidence rates. Although we performed manual correction and de-duplication, there may be records of target drugs that were not included. Secondly, although the use of disproportionality analysis in pharmacovigilance is well established, a limitation of such methods is the lack of a gold standard for assessing the validity and magnitude of suspected safety issues (Almenoff et al., 2007). We defined graded thresholds of signal intensity that allow clinicians to understand suspicious events more intuitively and quickly. Moreover, the significant signals monitored only represent potential associations rather than causality. Finally, we only analyzed the single and combined usage of SGLT2i, without comparing other hypoglycemic agents. This will be considered as our sequential study. Nevertheless, FAERS has provided us with a huge amount of drug safety data for free. We have systematically mined and identified potential IME signals associated with SGLT2is, as well as provided a signaling profile of SGLT2i-induced IMEs, which could provide valuable evidence for further research and clinical practice in this area.

## Conclusion

With the widespread clinical use of SGLT2i, concerns about safety issues have arisen. This study comprehensively explored the potential IME signals of SGLT2i based on all other drugs and other antidiabetic drugs as non-cases. The type and magnitude of IMEs varied among different SGLT2is. Except for cellulitis, osteomyelitis, and some cardiac/cerebral events, most results were consistent with previous studies. Our results can only indicate that these IMEs are overreported in SGLT2i, while proof of causality requires additional evidence, such as pharmacoepidemiological studies, pharmacokinetic and pharmacodynamics plausibility, or from pharmacological properties and pathophysiology.

## Data Availability

Publicly available datasets were analyzed in this study. This data can be found here: https://fis.fda.gov/extensions/FPD-QDE-FAERS/FPD-QDE-FAERS.html.
