# Supplementary material for: Safety of SGLT2 Inhibitors: A Pharmacovigilance Study from 2013 to 2021 Based on FAERS
Source: Front Pharmacol. 2021 Dec 20;12:766125. doi: 10.3389/fphar.2021.766125 (PMC8721280; doi:10.3389/fphar.2021.766125)
Supplement: Supplementary file 1 [file DataSheet1.docx]

**Supplementary Material**

# Table S1. Generic names and brand names of SGLT2i

| Generic name | Brand name |
| --- | --- |
| Canagliflozin | Canaglu^®^, Invokana^®^ |
| Canagliflozin/metformin hydrochloride | Invokana Duo^®^, Invokamet^®^, Vokanamet^®^ |
| Empagliflozin | Jardiance^®^ |
| Empagliflozin/metformin hydrochloride | Jardiamet^®^, Jardiance Duo^®^, Synjardy^®^ |
| Empagliflozin/linagliptin | Glyxambi^®^, Jardianz DPP^®^ |
| Empagliflozin/linagliptin/metformin | TRIJARDY XR^®^ |
| Dapagliflozin propanediol | Edistride^®^, Farxiga^®^, Forxiga^®^, Forziga^®^ |
| Dapagliflozin propanediol/metformin hydrochloride | Ebymect^®^, Xigduo^®^, Xigduo XR^®^ |
| Ertugliflozin | Steglatro^®^ |
| Ertugliflozin/metformin hydrochloride | Segluromet^®^ |
| Ertugliflozin/sitagliptin | Steglujan^®^ |
| Ipragliflozin | Suglat^®^ |
| Tofogliflozin | Apleway^®^, Deberza^®^ |
| Luseogliflozin | Lusefi^®^ |
| Remogliflozin etabonate | Remo^®^ |
| Sotagliflozin | Zynquista^®^ |

# Table S2. Two-by-two contingency table for the disproportionality analysis*

|  | Target AEs | Other AEs |
| --- | --- | --- |
| Target drugs | a | b |
| Other drugs | c | d |

*In Table S2, AEs: adverse effects; a is the number of reports of interested drug-AE pairs; b is the number of reports with all other AEs of the target drugs; c is the number of reports with the target AEs of all other drugs; d is the number of reports with all other AEs of all other drugs; In FAERS, all AEs are coded as Preferred Terms (PTs) according to MedDRA.We matched all PTs with the corresponding primary system organ classes (SOCs) and subsequently performed disproportionality analysis from the level of SOCs. At SOC level, a is the number of reports of interested drug-SOC pairs; b is the number of reports with all other SOCs of the target drugs; c is the number of reports with the target SOCs of all other drugs; d is the number of reports with all other SOCs of all other drugs


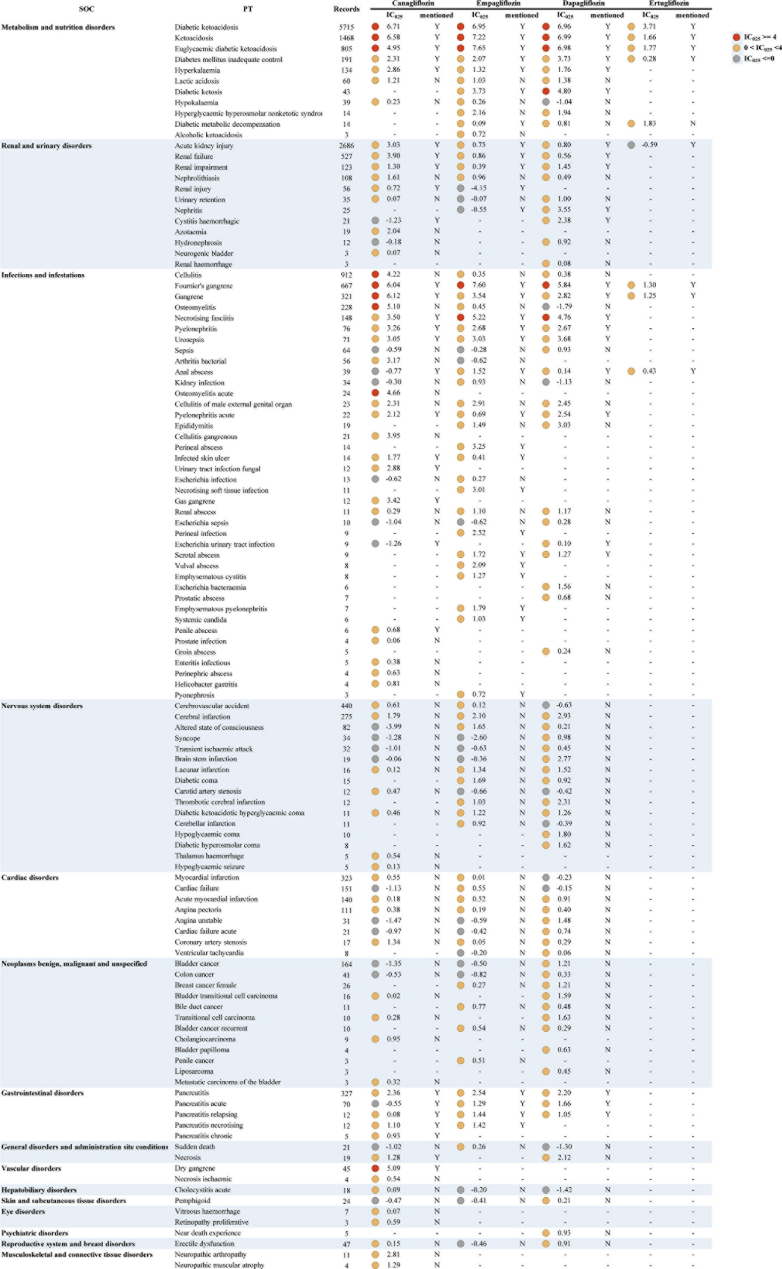


# Figure S1. Signal profiles of all IMEs induced by SGLT2i monotherapy-based on all other drugs as "non-case". *

*In Fig.S1, IMEs: Important Medical Events; SOC: System Organ Class; PT: Preferred Term; IC: information component; IC025: the lower limit of the 95% confidence interval of IC. IC025 greater than 0 was deemed a signal. IC025 no less than 4 was deemed a strong signal.

# Table S3. Disproportionality analysis results of all SGLT2i-related important medical events in monotherapy-based on all other drugs as "non-case".*

| Drugname | pt | a | b | c | d | IC | IC_025_ | IC_975_ | ROR | ROR_025_ | ROR_975_ |
| --- | --- | --- | --- | --- | --- | --- | --- | --- | --- | --- | --- |
| Canagliflozin | Diabetic ketoacidosis | 2393 | 22724 | 7615 | 11790097 | 6.78 | **6.71** | 6.83 | 109.99 | **104.86** | 115.37 |
|  | Acute kidney injury | 2127 | 22990 | 113990 | 11683722 | 3.11 | **3.03** | 3.16 | 8.61 | **8.23** | 9.00 |
|  | Cellulitis | 825 | 24292 | 18196 | 11779516 | 4.33 | **4.22** | 4.42 | 20.18 | **18.80** | 21.66 |
|  | Ketoacidosis | 524 | 24593 | 1576 | 11796136 | 6.72 | **6.58** | 6.83 | 105.72 | **95.69** | 116.79 |
|  | Renal failure | 451 | 24666 | 12129 | 11785583 | 4.05 | **3.90** | 4.16 | 16.58 | **15.08** | 18.23 |
|  | Gangrene | 258 | 24859 | 1026 | 11796686 | 6.32 | **6.12** | 6.47 | 80.09 | **69.83** | 91.85 |
|  | Cerebrovascular accident | 253 | 24864 | 67298 | 11730414 | 0.82 | **0.61** | 0.97 | 1.76 | **1.55** | 1.99 |
|  | Osteomyelitis | 215 | 24902 | 2076 | 11795636 | 5.33 | **5.10** | 5.49 | 40.15 | **34.87** | 46.23 |
|  | Fournier’s gangrene | 185 | 24932 | 701 | 11797011 | 6.28 | **6.04** | 6.46 | 77.87 | **66.19** | 91.60 |
|  | Myocardial infarction | 179 | 24938 | 48190 | 11749522 | 0.80 | **0.55** | 0.98 | 1.74 | **1.50** | 2.01 |
|  | Pancreatitis | 145 | 24972 | 10612 | 11787100 | 2.64 | **2.36** | 2.84 | 6.23 | **5.29** | 7.34 |
|  | Death | 132 | 24985 | 418646 | 11379066 | -2.75 | -3.04 | -2.54 | 0.15 | 0.13 | 0.18 |
|  | Euglycaemic diabetic ketoacidosis | 109 | 25008 | 994 | 11796718 | 5.27 | **4.95** | 5.50 | 38.51 | **31.59** | 46.95 |
|  | Cerebral infarction | 94 | 25023 | 9809 | 11787903 | 2.13 | **1.79** | 2.38 | 4.39 | **3.58** | 5.38 |
|  | Hyperkalaemia | 85 | 25032 | 4002 | 11793710 | 3.22 | **2.86** | 3.48 | 9.31 | **7.51** | 11.55 |
|  | Atrial fibrillation | 69 | 25048 | 39527 | 11758185 | -0.28 | -0.68 | 0.00 | 0.82 | 0.65 | 1.04 |
|  | Diabetes mellitus inadequate control | 64 | 25053 | 4292 | 11793420 | 2.73 | **2.31** | 3.02 | 6.61 | **5.16** | 8.47 |
|  | Renal impairment | 61 | 25056 | 8469 | 11789243 | 1.72 | **1.30** | 2.03 | 3.30 | **2.57** | 4.25 |
|  | Nephrolithiasis | 61 | 25056 | 6753 | 11790959 | 2.04 | **1.61** | 2.34 | 4.11 | **3.19** | 5.29 |
|  | Chronic kidney disease | 60 | 25057 | 52129 | 11745583 | -0.88 | -1.31 | -0.57 | 0.54 | 0.42 | 0.70 |
|  | Acute myocardial infarction | 52 | 25065 | 15533 | 11782179 | 0.64 | **0.18** | 0.97 | 1.56 | **1.19** | 2.05 |
|  | Renal injury | 51 | 25066 | 10347 | 11787365 | 1.19 | **0.72** | 1.52 | 2.28 | **1.73** | 3.00 |
|  | Arthritis bacterial | 51 | 25066 | 1667 | 11796045 | 3.63 | **3.17** | 3.97 | 12.41 | **9.39** | 16.40 |
|  | Angina pectoris | 50 | 25067 | 12858 | 11784854 | 0.85 | **0.38** | 1.19 | 1.81 | **1.37** | 2.39 |
|  | Dry gangrene | 42 | 25075 | 133 | 11797579 | 5.61 | **5.09** | 5.97 | 48.75 | **34.45** | 68.98 |
|  | Cardiac failure | 39 | 25078 | 27813 | 11769899 | -0.60 | -1.13 | -0.22 | 0.66 | 0.48 | 0.91 |
|  | Pyelonephritis | 37 | 25080 | 985 | 11796727 | 3.81 | **3.26** | 4.20 | 14.04 | **10.11** | 19.50 |
|  | Bladder cancer | 35 | 25082 | 28574 | 11769138 | -0.79 | -1.35 | -0.39 | 0.58 | 0.42 | 0.81 |
|  | Necrotising fasciitis | 32 | 25085 | 633 | 11797079 | 4.09 | **3.50** | 4.51 | 16.99 | **11.91** | 24.24 |
|  | Angioedema | 27 | 25090 | 21502 | 11776210 | -0.75 | -1.39 | -0.30 | 0.59 | 0.41 | 0.87 |
|  | Urosepsis | 26 | 25091 | 695 | 11797017 | 3.71 | **3.05** | 4.17 | 13.04 | **8.82** | 19.30 |
|  | Lactic acidosis | 26 | 25091 | 3154 | 11794558 | 1.87 | **1.21** | 2.33 | 3.65 | **2.48** | 5.37 |
|  | Arrhythmia | 24 | 25093 | 19445 | 11778267 | -0.77 | -1.46 | -0.29 | 0.59 | 0.39 | 0.87 |
|  | Osteomyelitis acute | 23 | 25094 | 12 | 11797700 | 5.35 | **4.66** | 5.85 | 40.92 | **20.36** | 82.24 |
|  | Cataract | 23 | 25094 | 20082 | 11777630 | -0.88 | -1.58 | -0.39 | 0.54 | 0.36 | 0.82 |
|  | Pneumonia | 21 | 25096 | 49768 | 11747944 | -2.31 | -3.04 | -1.79 | 0.20 | 0.13 | 0.31 |
|  | Cardiac failure congestive | 21 | 25096 | 33864 | 11763848 | -1.75 | -2.49 | -1.24 | 0.30 | 0.19 | 0.46 |
|  | Sepsis | 20 | 25097 | 8382 | 11789330 | 0.16 | -0.59 | 0.68 | 1.12 | 0.72 | 1.73 |
|  | Erectile dysfunction | 19 | 25098 | 4593 | 11793119 | 0.92 | **0.15** | 1.46 | 1.89 | **1.21** | 2.97 |
|  | Hypokalaemia | 19 | 25098 | 4324 | 11793388 | 1.00 | **0.23** | 1.54 | 2.00 | **1.28** | 3.15 |
|  | Loss of consciousness | 18 | 25099 | 8836 | 11788876 | -0.06 | -0.85 | 0.49 | 0.96 | 0.60 | 1.52 |
|  | Azotaemia | 18 | 25099 | 966 | 11796746 | 2.84 | **2.04** | 3.39 | 7.14 | **4.48** | 11.39 |
|  | Diabetes mellitus | 17 | 25100 | 17117 | 11780595 | -1.08 | -1.89 | -0.51 | 0.47 | 0.29 | 0.76 |
|  | Cellulitis gangrenous | 17 | 25100 | 51 | 11797661 | 4.76 | **3.95** | 5.33 | 27.15 | **15.68** | 47.02 |
|  | Anaphylactic reaction | 17 | 25100 | 25298 | 11772414 | -1.63 | -2.45 | -1.07 | 0.32 | 0.20 | 0.52 |
|  | Colon cancer | 16 | 25101 | 5986 | 11791726 | 0.32 | -0.53 | 0.90 | 1.25 | 0.76 | 2.03 |
|  | Cardiac arrest | 16 | 25101 | 26782 | 11770930 | -1.80 | -2.64 | -1.22 | 0.29 | 0.18 | 0.47 |
|  | Deep vein thrombosis | 16 | 25101 | 23640 | 11774072 | -1.62 | -2.46 | -1.04 | 0.33 | 0.20 | 0.53 |
|  | Acute respiratory failure | 16 | 25101 | 8410 | 11789302 | -0.16 | -1.00 | 0.43 | 0.90 | 0.55 | 1.46 |
|  | Hepatic cancer | 15 | 25102 | 7102 | 11790610 | -0.01 | -0.88 | 0.59 | 0.99 | 0.60 | 1.65 |
|  | Neoplasm malignant | 14 | 25103 | 16341 | 11781371 | -1.28 | -2.18 | -0.66 | 0.41 | 0.24 | 0.69 |
|  | Pancreatitis acute | 14 | 25103 | 5092 | 11792620 | 0.35 | -0.55 | 0.97 | 1.28 | 0.76 | 2.16 |
|  | Urinary retention | 13 | 25104 | 2904 | 11794808 | 1.01 | **0.07** | 1.65 | 2.02 | **1.17** | 3.48 |
|  | Cerebral haemorrhage | 13 | 25104 | 17062 | 11780650 | -1.45 | -2.38 | -0.80 | 0.37 | 0.21 | 0.63 |
|  | Renal cancer | 12 | 25105 | 16211 | 11781501 | -1.48 | -2.46 | -0.82 | 0.36 | 0.20 | 0.63 |
|  | Kidney infection | 12 | 25105 | 3423 | 11794289 | 0.68 | -0.30 | 1.35 | 1.60 | 0.91 | 2.83 |
|  | Gas gangrene | 12 | 25105 | 31 | 11797681 | 4.40 | **3.42** | 5.07 | 21.14 | **10.85** | 41.16 |
|  | Gastric cancer | 12 | 25105 | 8757 | 11788955 | -0.61 | -1.59 | 0.05 | 0.65 | 0.37 | 1.15 |
|  | Bacteraemia | 12 | 25105 | 4166 | 11793546 | 0.41 | -0.56 | 1.08 | 1.33 | 0.76 | 2.35 |
|  | Erythema multiforme | 11 | 25106 | 2819 | 11794893 | 0.82 | -0.20 | 1.52 | 1.77 | 0.98 | 3.19 |
|  | Breast cancer | 11 | 25106 | 33243 | 11764469 | -2.63 | -3.65 | -1.93 | 0.16 | 0.09 | 0.29 |
|  | Transient ischaemic attack | 10 | 25107 | 4458 | 11793254 | 0.07 | -1.01 | 0.80 | 1.05 | 0.56 | 1.95 |
|  | Syncope | 10 | 25107 | 5458 | 11792254 | -0.21 | -1.28 | 0.52 | 0.87 | 0.47 | 1.61 |
|  | Prostate cancer | 10 | 25107 | 21860 | 11775852 | -2.16 | -3.24 | -1.44 | 0.22 | 0.12 | 0.42 |
|  | Acute coronary syndrome | 10 | 25107 | 4846 | 11792866 | -0.04 | -1.12 | 0.68 | 0.97 | 0.52 | 1.81 |
|  | Blindness | 10 | 25107 | 16982 | 11780730 | -1.80 | -2.88 | -1.08 | 0.29 | 0.15 | 0.53 |
|  | Suicidal ideation | 9 | 25108 | 5221 | 11792491 | -0.29 | -1.43 | 0.47 | 0.82 | 0.43 | 1.57 |
|  | Seizure | 9 | 25108 | 17041 | 11780671 | -1.95 | -3.09 | -1.19 | 0.26 | 0.13 | 0.50 |
|  | Urinary tract infection fungal | 9 | 25108 | 32 | 11797680 | 4.02 | **2.88** | 4.78 | 16.18 | **7.72** | 33.90 |
|  | Pyelonephritis acute | 9 | 25108 | 223 | 11797489 | 3.26 | **2.12** | 4.02 | 9.57 | **4.91** | 18.63 |
|  | Pemphigoid | 9 | 25108 | 2561 | 11795151 | 0.67 | -0.47 | 1.43 | 1.59 | 0.83 | 3.07 |
|  | Neuropathic arthropathy | 9 | 25108 | 46 | 11797666 | 3.94 | **2.81** | 4.71 | 15.40 | **7.54** | 31.47 |
|  | Neuropathy peripheral | 9 | 25108 | 12901 | 11784811 | -1.56 | -2.69 | -0.79 | 0.34 | 0.18 | 0.65 |
|  | Pancreatic carcinoma | 9 | 25108 | 9943 | 11787769 | -1.19 | -2.33 | -0.43 | 0.44 | 0.23 | 0.84 |
|  | Hepatic cirrhosis | 9 | 25108 | 3206 | 11794506 | 0.37 | -0.76 | 1.14 | 1.30 | 0.67 | 2.49 |
|  | Disability | 9 | 25108 | 5555 | 11792157 | -0.38 | -1.51 | 0.39 | 0.77 | 0.40 | 1.48 |
|  | Infected skin ulcer | 9 | 25108 | 350 | 11797362 | 2.91 | **1.77** | 3.67 | 7.52 | **3.88** | 14.58 |
|  | Cholecystitis acute | 9 | 25108 | 1661 | 11796051 | 1.23 | **0.09** | 1.99 | 2.35 | **1.22** | 4.52 |
|  | Coronary artery stenosis | 9 | 25108 | 555 | 11797157 | 2.48 | **1.34** | 3.25 | 5.59 | **2.90** | 10.81 |
|  | Myocardial ischaemia | 8 | 25109 | 1754 | 11795958 | 1.00 | -0.21 | 1.81 | 2.00 | **1.00** | 4.01 |
|  | Necrosis | 8 | 25109 | 466 | 11797246 | 2.50 | **1.28** | 3.30 | 5.64 | **2.80** | 11.35 |
|  | Ischaemic stroke | 8 | 25109 | 5458 | 11792254 | -0.51 | -1.72 | 0.29 | 0.70 | 0.35 | 1.40 |
|  | Coma | 8 | 25109 | 12238 | 11785474 | -1.64 | -2.85 | -0.84 | 0.32 | 0.16 | 0.64 |
|  | Cholangiocarcinoma | 8 | 25109 | 652 | 11797060 | 2.16 | **0.95** | 2.96 | 4.47 | **2.23** | 8.97 |
|  | Cardio-respiratory arrest | 8 | 25109 | 14741 | 11782971 | -1.91 | -3.12 | -1.10 | 0.27 | 0.13 | 0.53 |
|  | Anal abscess | 8 | 25109 | 2706 | 11795006 | 0.44 | -0.77 | 1.24 | 1.36 | 0.68 | 2.72 |
|  | Dementia Alzheimers type | 8 | 25109 | 2735 | 11794977 | 0.43 | -0.79 | 1.23 | 1.34 | 0.67 | 2.69 |
|  | Sudden death | 7 | 25110 | 2657 | 11795055 | 0.28 | -1.02 | 1.14 | 1.22 | 0.58 | 2.56 |
|  | Haematochezia | 7 | 25110 | 5435 | 11792277 | -0.69 | -1.99 | 0.17 | 0.62 | 0.30 | 1.31 |
|  | Facial paralysis | 7 | 25110 | 2195 | 11795517 | 0.53 | -0.77 | 1.39 | 1.45 | 0.69 | 3.04 |
|  | Hallucination | 7 | 25110 | 9192 | 11788520 | -1.42 | -2.72 | -0.57 | 0.37 | 0.18 | 0.79 |
|  | Hepatitis | 7 | 25110 | 5008 | 11792704 | -0.57 | -1.87 | 0.28 | 0.67 | 0.32 | 1.41 |
|  | Lung neoplasm malignant | 7 | 25110 | 10905 | 11786807 | -1.66 | -2.96 | -0.81 | 0.32 | 0.15 | 0.66 |
|  | Intestinal obstruction | 7 | 25110 | 6467 | 11791245 | -0.93 | -2.23 | -0.07 | 0.53 | 0.25 | 1.10 |
|  | Glaucoma | 7 | 25110 | 3564 | 11794148 | -0.11 | -1.41 | 0.74 | 0.93 | 0.44 | 1.95 |
|  | Circulatory collapse | 7 | 25110 | 3991 | 11793721 | -0.26 | -1.56 | 0.59 | 0.83 | 0.40 | 1.75 |
|  | Choking | 7 | 25110 | 6792 | 11790920 | -0.99 | -2.30 | -0.14 | 0.50 | 0.24 | 1.05 |
|  | Abdominal infection | 7 | 25110 | 1399 | 11796313 | 1.10 | -0.20 | 1.96 | 2.15 | **1.02** | 4.52 |
|  | Cellulitis of male external genital organ | 7 | 25110 | 46 | 11797666 | 3.61 | **2.31** | 4.47 | 12.24 | **5.53** | 27.12 |
|  | Pulmonary embolism | 6 | 25111 | 14667 | 11783045 | -2.28 | -3.70 | -1.37 | 0.21 | 0.09 | 0.46 |
|  | Pancreatitis necrotising | 6 | 25111 | 294 | 11797418 | 2.51 | **1.10** | 3.43 | 5.72 | **2.55** | 12.83 |
|  | Retinal haemorrhage | 6 | 25111 | 1019 | 11796693 | 1.28 | -0.14 | 2.19 | 2.43 | **1.09** | 5.42 |
|  | Rhabdomyolysis | 6 | 25111 | 3334 | 11794378 | -0.22 | -1.64 | 0.69 | 0.86 | 0.38 | 1.91 |
|  | Diverticulitis | 6 | 25111 | 7511 | 11790201 | -1.34 | -2.76 | -0.43 | 0.39 | 0.18 | 0.88 |
|  | Device related infection | 6 | 25111 | 5166 | 11792546 | -0.82 | -2.24 | 0.09 | 0.57 | 0.25 | 1.26 |
|  | Drug-induced liver injury | 6 | 25111 | 9757 | 11787955 | -1.71 | -3.12 | -0.80 | 0.31 | 0.14 | 0.68 |
|  | Haemorrhage | 6 | 25111 | 24057 | 11773655 | -2.99 | -4.40 | -2.08 | 0.13 | 0.06 | 0.28 |
|  | Liver injury | 6 | 25111 | 3178 | 11794534 | -0.16 | -1.58 | 0.75 | 0.89 | 0.40 | 1.99 |
|  | Bladder transitional cell carcinoma | 6 | 25111 | 894 | 11796818 | 1.43 | **0.02** | 2.34 | 2.69 | **1.21** | 6.02 |
|  | Angina unstable | 6 | 25111 | 2948 | 11794764 | -0.06 | -1.47 | 0.85 | 0.96 | 0.43 | 2.14 |
|  | Atrioventricular block complete | 6 | 25111 | 2558 | 11795154 | 0.13 | -1.29 | 1.04 | 1.09 | 0.49 | 2.44 |
|  | Cardiac failure acute | 6 | 25111 | 2001 | 11795711 | 0.45 | -0.97 | 1.36 | 1.36 | 0.61 | 3.04 |
|  | Cholecystitis | 6 | 25111 | 3385 | 11794327 | -0.25 | -1.66 | 0.67 | 0.84 | 0.38 | 1.88 |
|  | Bradycardia | 6 | 25111 | 16955 | 11780757 | -2.49 | -3.91 | -1.58 | 0.18 | 0.08 | 0.40 |
|  | Carotid artery stenosis | 6 | 25111 | 588 | 11797124 | 1.88 | **0.47** | 2.79 | 3.69 | **1.65** | 8.25 |
|  | Cachexia | 6 | 25111 | 1141 | 11796571 | 1.15 | -0.27 | 2.06 | 2.21 | 0.99 | 4.94 |
|  | Pancreatitis chronic | 5 | 25112 | 221 | 11797491 | 2.49 | **0.93** | 3.47 | 5.61 | **2.31** | 13.62 |
|  | Stevens-Johnson syndrome | 5 | 25112 | 3284 | 11794428 | -0.45 | -2.01 | 0.54 | 0.73 | 0.31 | 1.77 |
|  | Retinal detachment | 5 | 25112 | 1499 | 11796213 | 0.57 | -0.99 | 1.56 | 1.49 | 0.62 | 3.58 |
|  | Rectal haemorrhage | 5 | 25112 | 4726 | 11792986 | -0.94 | -2.50 | 0.04 | 0.52 | 0.22 | 1.25 |
|  | Interstitial lung disease | 5 | 25112 | 13087 | 11784625 | -2.36 | -3.93 | -1.38 | 0.19 | 0.08 | 0.47 |
|  | Escherichia infection | 5 | 25112 | 1104 | 11796608 | 0.95 | -0.62 | 1.93 | 1.93 | 0.80 | 4.64 |
|  | Femur fracture | 5 | 25112 | 5618 | 11792094 | -1.18 | -2.74 | -0.19 | 0.44 | 0.18 | 1.06 |
|  | Hydronephrosis | 5 | 25112 | 756 | 11796956 | 1.38 | -0.18 | 2.36 | 2.60 | **1.08** | 6.26 |
|  | Enteritis infectious | 5 | 25112 | 433 | 11797279 | 1.94 | **0.38** | 2.93 | 3.84 | **1.59** | 9.28 |
|  | Helicobacter infection | 5 | 25112 | 651 | 11797061 | 1.54 | -0.02 | 2.52 | 2.90 | **1.20** | 7.00 |
|  | Hepatic failure | 5 | 25112 | 5426 | 11792286 | -1.13 | -2.69 | -0.15 | 0.46 | 0.19 | 1.10 |
|  | Hepatocellular carcinoma | 5 | 25112 | 3155 | 11794557 | -0.39 | -1.95 | 0.59 | 0.76 | 0.32 | 1.83 |
|  | Appendicitis | 5 | 25112 | 3757 | 11793955 | -0.63 | -2.19 | 0.36 | 0.65 | 0.27 | 1.56 |
|  | Apparent death | 5 | 25112 | 2612 | 11795100 | -0.14 | -1.70 | 0.84 | 0.91 | 0.38 | 2.18 |
|  | Thrombosis | 4 | 25113 | 11597 | 11786115 | -2.48 | -4.25 | -1.40 | 0.18 | 0.07 | 0.48 |
|  | Subarachnoid haemorrhage | 4 | 25113 | 1267 | 11796445 | 0.49 | -1.27 | 1.57 | 1.41 | 0.53 | 3.75 |
|  | Toxic skin eruption | 4 | 25113 | 1778 | 11795934 | 0.07 | -1.69 | 1.15 | 1.05 | 0.39 | 2.80 |
|  | Renal cell carcinoma | 4 | 25113 | 603 | 11797109 | 1.33 | -0.43 | 2.41 | 2.51 | 0.94 | 6.72 |
|  | Thalamus haemorrhage | 4 | 25113 | 189 | 11797523 | 2.31 | **0.54** | 3.38 | 4.94 | **1.84** | 13.31 |
|  | Oesophageal carcinoma | 4 | 25113 | 8349 | 11789363 | -2.02 | -3.78 | -0.94 | 0.25 | 0.09 | 0.66 |
|  | Toxic epidermal necrolysis | 4 | 25113 | 2010 | 11795702 | -0.09 | -1.85 | 0.99 | 0.94 | 0.35 | 2.51 |
|  | Vitreous haemorrhage | 4 | 25113 | 355 | 11797357 | 1.83 | **0.07** | 2.91 | 3.56 | **1.33** | 9.55 |
|  | Transitional cell carcinoma | 4 | 25113 | 273 | 11797439 | 2.05 | **0.28** | 3.13 | 4.13 | **1.54** | 11.10 |
|  | Neuropathic muscular atrophy | 4 | 25113 | 16 | 11797696 | 3.05 | **1.29** | 4.13 | 8.30 | **2.77** | 24.81 |
|  | Renal tubular necrosis | 4 | 25113 | 493 | 11797219 | 1.53 | -0.23 | 2.61 | 2.89 | **1.08** | 7.74 |
|  | Lacunar infarction | 4 | 25113 | 334 | 11797378 | 1.89 | **0.12** | 2.96 | 3.69 | **1.38** | 9.90 |
|  | Hip fracture | 4 | 25113 | 4093 | 11793619 | -1.03 | -2.80 | 0.05 | 0.49 | 0.18 | 1.30 |
|  | Gastric ulcer | 4 | 25113 | 3388 | 11794324 | -0.78 | -2.54 | 0.30 | 0.58 | 0.22 | 1.56 |
|  | Epilepsy | 4 | 25113 | 7409 | 11790303 | -1.85 | -3.62 | -0.77 | 0.28 | 0.10 | 0.74 |
|  | Helicobacter gastritis | 4 | 25113 | 116 | 11797596 | 2.58 | **0.81** | 3.65 | 5.96 | **2.20** | 16.15 |
|  | Hypersensitivity vasculitis | 4 | 25113 | 1581 | 11796131 | 0.22 | -1.55 | 1.30 | 1.16 | 0.44 | 3.10 |
|  | Coeliac disease | 4 | 25113 | 3644 | 11794068 | -0.87 | -2.64 | 0.20 | 0.55 | 0.20 | 1.45 |
|  | Bile duct stone | 4 | 25113 | 1175 | 11796537 | 0.58 | -1.18 | 1.66 | 1.50 | 0.56 | 4.00 |
|  | Cystitis haemorrhagic | 4 | 25113 | 1221 | 11796491 | 0.54 | -1.23 | 1.62 | 1.45 | 0.54 | 3.87 |
|  | Abortion spontaneous | 4 | 25113 | 22957 | 11774755 | -3.45 | -5.22 | -2.37 | 0.09 | 0.03 | 0.24 |
|  | Acute respiratory distress syndrome | 4 | 25113 | 7139 | 11790573 | -1.80 | -3.57 | -0.72 | 0.29 | 0.11 | 0.77 |
|  | Cervix carcinoma | 4 | 25113 | 1562 | 11796150 | 0.23 | -1.53 | 1.31 | 1.18 | 0.44 | 3.14 |
|  | Colitis ischaemic | 4 | 25113 | 2226 | 11795486 | -0.22 | -1.98 | 0.86 | 0.86 | 0.32 | 2.29 |
|  | Completed suicide | 4 | 25113 | 33712 | 11764000 | -4.00 | -5.77 | -2.92 | 0.06 | 0.02 | 0.17 |
|  | Blindness transient | 4 | 25113 | 2974 | 11794738 | -0.60 | -2.37 | 0.48 | 0.66 | 0.25 | 1.76 |
|  | Brain stem infarction | 4 | 25113 | 410 | 11797302 | 1.71 | -0.06 | 2.78 | 3.26 | **1.22** | 8.73 |
|  | Altered state of consciousness | 4 | 25113 | 9629 | 11788083 | -2.22 | -3.99 | -1.14 | 0.21 | 0.08 | 0.57 |
|  | Acute hepatic failure | 4 | 25113 | 6942 | 11790770 | -1.76 | -3.53 | -0.68 | 0.29 | 0.11 | 0.79 |
|  | Blindness unilateral | 4 | 25113 | 5868 | 11791844 | -1.53 | -3.29 | -0.45 | 0.35 | 0.13 | 0.92 |
|  | Type 1 diabetes mellitus | 3 | 25114 | 557 | 11797155 | 1.05 | -1.02 | 2.26 | 2.07 | 0.67 | 6.44 |
|  | Sudden hearing loss | 3 | 25114 | 502 | 11797210 | 1.15 | -0.92 | 2.36 | 2.23 | 0.72 | 6.92 |
|  | Septic shock | 3 | 25114 | 1874 | 11795838 | -0.36 | -2.43 | 0.85 | 0.78 | 0.25 | 2.42 |
|  | Prostate infection | 3 | 25114 | 138 | 11797574 | 2.13 | **0.06** | 3.34 | 4.38 | **1.39** | 13.74 |
|  | Perinephric abscess | 3 | 25114 | 16 | 11797696 | 2.70 | **0.63** | 3.90 | 6.48 | **1.89** | 22.23 |
|  | Rectal cancer | 3 | 25114 | 652 | 11797060 | 0.89 | -1.18 | 2.09 | 1.85 | 0.60 | 5.75 |
|  | Pancreatitis relapsing | 3 | 25114 | 133 | 11797579 | 2.15 | **0.08** | 3.36 | 4.44 | **1.41** | 13.93 |
|  | Renal abscess | 3 | 25114 | 82 | 11797630 | 2.36 | **0.29** | 3.57 | 5.14 | **1.62** | 16.28 |
|  | Penile abscess | 3 | 25114 | 6 | 11797706 | 2.75 | **0.68** | 3.96 | 6.74 | **1.69** | 26.96 |
|  | Nephrotic syndrome | 3 | 25114 | 1068 | 11796644 | 0.33 | -1.73 | 1.54 | 1.26 | 0.41 | 3.92 |
|  | Thrombocytopenia | 3 | 25114 | 6933 | 11790779 | -2.12 | -4.19 | -0.92 | 0.23 | 0.07 | 0.71 |
|  | Neurogenic bladder | 3 | 25114 | 136 | 11797576 | 2.14 | **0.07** | 3.34 | 4.40 | **1.40** | 13.82 |
|  | Peripheral artery occlusion | 3 | 25114 | 191 | 11797521 | 1.94 | -0.13 | 3.15 | 3.84 | **1.23** | 12.00 |
|  | Renal tubular acidosis | 3 | 25114 | 228 | 11797484 | 1.82 | -0.25 | 3.03 | 3.53 | **1.13** | 11.04 |
|  | Necrosis ischaemic | 3 | 25114 | 32 | 11797680 | 2.61 | **0.54** | 3.81 | 6.09 | **1.87** | 19.90 |
|  | Retinopathy proliferative | 3 | 25114 | 22 | 11797690 | 2.66 | **0.59** | 3.87 | 6.33 | **1.89** | 21.14 |
|  | Diabetic ketoacidotic hyperglycaemic coma | 3 | 25114 | 47 | 11797665 | 2.53 | **0.46** | 3.74 | 5.77 | **1.80** | 18.55 |
|  | Enterocolitis | 3 | 25114 | 1453 | 11796259 | -0.04 | -2.11 | 1.17 | 0.97 | 0.31 | 3.02 |
|  | Gastritis erosive | 3 | 25114 | 557 | 11797155 | 1.05 | -1.02 | 2.26 | 2.07 | 0.67 | 6.44 |
|  | Escherichia sepsis | 3 | 25114 | 567 | 11797145 | 1.03 | -1.04 | 2.24 | 2.05 | 0.66 | 6.36 |
|  | Macular fibrosis | 3 | 25114 | 211 | 11797501 | 1.87 | -0.20 | 3.08 | 3.67 | **1.17** | 11.46 |
|  | Diplegia | 3 | 25114 | 501 | 11797211 | 1.16 | -0.91 | 2.36 | 2.23 | 0.72 | 6.93 |
|  | Escherichia urinary tract infection | 3 | 25114 | 701 | 11797011 | 0.81 | -1.26 | 2.02 | 1.75 | 0.56 | 5.45 |
|  | Metastatic carcinoma of the bladder | 3 | 25114 | 77 | 11797635 | 2.39 | **0.32** | 3.59 | 5.22 | **1.65** | 16.56 |
|  | Hypoglycaemic seizure | 3 | 25114 | 120 | 11797592 | 2.20 | **0.13** | 3.41 | 4.60 | **1.46** | 14.46 |
|  | Ischaemic cerebral infarction | 3 | 25114 | 228 | 11797484 | 1.82 | -0.25 | 3.03 | 3.53 | **1.13** | 11.04 |
|  | Disseminated intravascular coagulation | 3 | 25114 | 2582 | 11795130 | -0.78 | -2.84 | 0.43 | 0.58 | 0.19 | 1.81 |
|  | Haematemesis | 3 | 25114 | 3363 | 11794349 | -1.13 | -3.20 | 0.08 | 0.46 | 0.15 | 1.42 |
|  | Hypovolaemic shock | 3 | 25114 | 411 | 11797301 | 1.34 | -0.73 | 2.55 | 2.54 | 0.81 | 7.90 |
|  | Gallbladder cancer | 3 | 25114 | 389 | 11797323 | 1.39 | -0.68 | 2.60 | 2.63 | 0.84 | 8.18 |
|  | Heart injury | 3 | 25114 | 204 | 11797508 | 1.90 | -0.17 | 3.10 | 3.72 | **1.19** | 11.64 |
|  | Accidental death | 3 | 25114 | 773 | 11796939 | 0.70 | -1.37 | 1.91 | 1.63 | 0.52 | 5.06 |
|  | Cutaneous vasculitis | 3 | 25114 | 544 | 11797168 | 1.07 | -0.99 | 2.28 | 2.11 | 0.68 | 6.55 |
|  | Adenocarcinoma of colon | 3 | 25114 | 1378 | 11796334 | 0.03 | -2.04 | 1.23 | 1.02 | 0.33 | 3.16 |
|  | Beta haemolytic streptococcal infection | 3 | 25114 | 398 | 11797314 | 1.37 | -0.70 | 2.58 | 2.59 | 0.83 | 8.06 |
|  | Basedows disease | 3 | 25114 | 1180 | 11796532 | 0.22 | -1.85 | 1.42 | 1.16 | 0.37 | 3.61 |
|  | Abdominal abscess | 3 | 25114 | 2774 | 11794938 | -0.87 | -2.94 | 0.34 | 0.55 | 0.18 | 1.70 |
|  | Bacterial sepsis | 3 | 25114 | 1057 | 11796655 | 0.35 | -1.72 | 1.55 | 1.27 | 0.41 | 3.95 |
|  | Cardiac flutter | 3 | 25114 | 1860 | 11795852 | -0.35 | -2.42 | 0.86 | 0.79 | 0.25 | 2.44 |
|  | Crohns disease | 3 | 25114 | 19586 | 11778126 | -3.59 | -5.66 | -2.38 | 0.08 | 0.03 | 0.26 |
|  | Candida sepsis | 3 | 25114 | 225 | 11797487 | 1.83 | -0.24 | 3.04 | 3.56 | **1.14** | 11.11 |
|  | Delirium | 3 | 25114 | 7195 | 11790517 | -2.17 | -4.24 | -0.97 | 0.22 | 0.07 | 0.69 |
|  | Aortic stenosis | 3 | 25114 | 1338 | 11796374 | 0.06 | -2.01 | 1.27 | 1.05 | 0.34 | 3.24 |
|  | Breast cancer recurrent | 3 | 25114 | 1052 | 11796660 | 0.35 | -1.72 | 1.56 | 1.28 | 0.41 | 3.97 |
|  | Bipolar I disorder | 3 | 25114 | 657 | 11797055 | 0.88 | -1.19 | 2.09 | 1.84 | 0.59 | 5.72 |
|  | Cellulitis staphylococcal | 3 | 25114 | 214 | 11797498 | 1.86 | -0.20 | 3.07 | 3.64 | **1.17** | 11.38 |
|  | Brain oedema | 3 | 25114 | 3274 | 11794438 | -1.09 | -3.16 | 0.11 | 0.47 | 0.15 | 1.46 |
|  | Brain injury | 3 | 25114 | 3804 | 11793908 | -1.29 | -3.36 | -0.09 | 0.41 | 0.13 | 1.26 |
| Empagliflozin | Diabetic ketoacidosis | 1864 | 14420 | 8144 | 11798401 | 7.03 | **6.95** | 7.08 | 130.53 | **123.80** | 137.62 |
|  | Ketoacidosis | 555 | 15729 | 1545 | 11805000 | 7.36 | **7.22** | 7.46 | 163.75 | **148.43** | 180.65 |
|  | Euglycaemic diabetic ketoacidosis | 451 | 15833 | 652 | 11805893 | 7.80 | **7.65** | 7.92 | 223.60 | **198.11** | 252.38 |
|  | Fournier’s gangrene | 376 | 15908 | 510 | 11806035 | 7.77 | **7.60** | 7.90 | 218.85 | **191.38** | 250.27 |
|  | Acute kidney injury | 307 | 15977 | 115810 | 11690735 | 0.94 | **0.75** | 1.08 | 1.92 | **1.71** | 2.15 |
|  | Cerebrovascular accident | 124 | 16160 | 67427 | 11739118 | 0.41 | **0.12** | 0.63 | 1.33 | **1.12** | 1.59 |
|  | Pancreatitis | 110 | 16174 | 10647 | 11795898 | 2.85 | **2.54** | 3.08 | 7.21 | **5.98** | 8.71 |
|  | Death | 97 | 16187 | 418681 | 11387864 | -2.57 | -2.90 | -2.32 | 0.17 | 0.14 | 0.21 |
|  | Myocardial infarction | 86 | 16198 | 48283 | 11758262 | 0.37 | **0.01** | 0.62 | 1.29 | **1.04** | 1.59 |
|  | Cerebral infarction | 78 | 16206 | 9825 | 11796720 | 2.47 | **2.10** | 2.74 | 5.55 | **4.44** | 6.94 |
|  | Cardiac failure | 74 | 16210 | 27778 | 11778767 | 0.94 | **0.55** | 1.22 | 1.92 | **1.53** | 2.41 |
|  | Necrotising fasciitis | 69 | 16215 | 596 | 11805949 | 5.62 | **5.22** | 5.90 | 49.08 | **38.24** | 63.01 |
|  | Altered state of consciousness | 58 | 16226 | 9575 | 11796970 | 2.09 | **1.65** | 2.40 | 4.25 | **3.28** | 5.50 |
|  | Atrial fibrillation | 51 | 16233 | 39545 | 11767000 | -0.10 | -0.56 | 0.24 | 0.94 | 0.71 | 1.23 |
|  | Cellulitis | 47 | 16237 | 18974 | 11787571 | 0.83 | **0.35** | 1.18 | 1.78 | **1.34** | 2.37 |
|  | Renal failure | 45 | 16239 | 12535 | 11794010 | 1.35 | **0.86** | 1.71 | 2.55 | **1.90** | 3.42 |
|  | Acute myocardial infarction | 44 | 16240 | 15541 | 11791004 | 1.02 | **0.52** | 1.38 | 2.03 | **1.51** | 2.72 |
|  | Bladder cancer | 40 | 16244 | 28569 | 11777976 | 0.02 | -0.50 | 0.40 | 1.01 | 0.74 | 1.38 |
|  | Diabetes mellitus inadequate control | 39 | 16245 | 4317 | 11802228 | 2.60 | **2.07** | 2.98 | 6.08 | **4.43** | 8.33 |
|  | Gangrene | 38 | 16246 | 1246 | 11805299 | 4.09 | **3.54** | 4.47 | 16.97 | **12.29** | 23.45 |
|  | Angina pectoris | 31 | 16253 | 12877 | 11793668 | 0.79 | **0.19** | 1.21 | 1.72 | **1.21** | 2.45 |
|  | Nephrolithiasis | 29 | 16255 | 6785 | 11799760 | 1.58 | **0.96** | 2.02 | 2.98 | **2.07** | 4.30 |
|  | Pancreatitis acute | 28 | 16256 | 5078 | 11801467 | 1.92 | **1.29** | 2.37 | 3.78 | **2.61** | 5.49 |
|  | Renal impairment | 25 | 16259 | 8505 | 11798040 | 1.06 | **0.39** | 1.53 | 2.08 | **1.41** | 3.08 |
|  | Hyperkalaemia | 24 | 16260 | 4063 | 11802482 | 2.00 | **1.32** | 2.48 | 4.00 | **2.68** | 5.97 |
|  | Angioedema | 23 | 16261 | 21506 | 11785039 | -0.36 | -1.06 | 0.13 | 0.78 | 0.52 | 1.17 |
|  | Cardiac failure congestive | 21 | 16263 | 33864 | 11772681 | -1.13 | -1.87 | -0.62 | 0.46 | 0.30 | 0.70 |
|  | Urosepsis | 20 | 16264 | 701 | 11805844 | 3.78 | **3.03** | 4.30 | 13.73 | **8.80** | 21.42 |
|  | Pyelonephritis | 20 | 16264 | 1002 | 11805543 | 3.43 | **2.68** | 3.95 | 10.75 | **6.90** | 16.73 |
|  | Anal abscess | 20 | 16264 | 2694 | 11803851 | 2.27 | **1.52** | 2.80 | 4.84 | **3.11** | 7.51 |
|  | Chronic kidney disease | 19 | 16265 | 52170 | 11754375 | -1.89 | -2.66 | -1.35 | 0.27 | 0.17 | 0.42 |
|  | Pancreatic carcinoma | 18 | 16266 | 9934 | 11796611 | 0.38 | -0.41 | 0.93 | 1.30 | 0.82 | 2.07 |
|  | Sepsis | 17 | 16267 | 8385 | 11798160 | 0.54 | -0.28 | 1.10 | 1.45 | 0.90 | 2.33 |
|  | Kidney infection | 17 | 16267 | 3418 | 11803127 | 1.74 | **0.93** | 2.31 | 3.35 | **2.08** | 5.39 |
|  | Lactic acidosis | 17 | 16267 | 3163 | 11803382 | 1.84 | **1.03** | 2.41 | 3.59 | **2.23** | 5.78 |
|  | Cardiac arrest | 16 | 16268 | 26782 | 11779763 | -1.18 | -2.02 | -0.60 | 0.44 | 0.27 | 0.72 |
|  | Deep vein thrombosis | 16 | 16268 | 23640 | 11782905 | -1.00 | -1.85 | -0.42 | 0.50 | 0.31 | 0.81 |
|  | Diabetic ketosis | 15 | 16269 | 86 | 11806459 | 4.60 | **3.73** | 5.20 | 24.25 | **14.01** | 41.98 |
|  | Diabetes mellitus | 15 | 16269 | 17119 | 11789426 | -0.64 | -1.51 | -0.04 | 0.64 | 0.39 | 1.07 |
|  | Coma | 15 | 16269 | 12231 | 11794314 | -0.16 | -1.04 | 0.44 | 0.89 | 0.54 | 1.48 |
|  | Neoplasm malignant | 14 | 16270 | 16341 | 11790204 | -0.67 | -1.57 | -0.05 | 0.63 | 0.37 | 1.06 |
|  | Pneumonia | 14 | 16270 | 49775 | 11756770 | -2.25 | -3.15 | -1.63 | 0.21 | 0.12 | 0.35 |
|  | Hypokalaemia | 14 | 16270 | 4329 | 11802216 | 1.16 | **0.26** | 1.78 | 2.24 | **1.32** | 3.78 |
|  | Loss of consciousness | 14 | 16270 | 8840 | 11797705 | 0.19 | -0.71 | 0.81 | 1.14 | 0.68 | 1.93 |
|  | Cataract | 14 | 16270 | 20091 | 11786454 | -0.96 | -1.86 | -0.34 | 0.51 | 0.30 | 0.87 |
|  | Arrhythmia | 13 | 16271 | 19456 | 11787089 | -1.02 | -1.96 | -0.37 | 0.49 | 0.29 | 0.85 |
|  | Ischaemic stroke | 12 | 16272 | 5454 | 11801091 | 0.64 | -0.34 | 1.31 | 1.56 | 0.88 | 2.74 |
|  | Delirium | 12 | 16272 | 7186 | 11799359 | 0.26 | -0.72 | 0.93 | 1.20 | 0.68 | 2.12 |
|  | Perineal abscess | 11 | 16273 | 57 | 11806488 | 4.28 | **3.25** | 4.97 | 19.37 | **10.16** | 36.95 |
|  | Lung neoplasm malignant | 11 | 16273 | 10901 | 11795644 | -0.43 | -1.46 | 0.26 | 0.74 | 0.41 | 1.34 |
|  | Breast cancer female | 11 | 16273 | 3025 | 11803520 | 1.30 | **0.27** | 1.99 | 2.46 | **1.36** | 4.44 |
|  | Blindness | 11 | 16273 | 16981 | 11789564 | -1.06 | -2.08 | -0.36 | 0.48 | 0.27 | 0.87 |
|  | Pulmonary embolism | 10 | 16274 | 14663 | 11791882 | -0.98 | -2.06 | -0.25 | 0.51 | 0.27 | 0.94 |
|  | Sudden death | 10 | 16274 | 2654 | 11803891 | 1.33 | **0.26** | 2.06 | 2.52 | **1.35** | 4.69 |
|  | Osteomyelitis | 10 | 16274 | 2281 | 11804264 | 1.52 | **0.45** | 2.25 | 2.87 | **1.54** | 5.35 |
|  | Disability | 10 | 16274 | 5554 | 11800991 | 0.36 | -0.71 | 1.09 | 1.29 | 0.69 | 2.39 |
|  | Erectile dysfunction | 10 | 16274 | 4602 | 11801943 | 0.62 | -0.46 | 1.34 | 1.53 | 0.82 | 2.85 |
|  | Colon cancer | 10 | 16274 | 5992 | 11800553 | 0.26 | -0.82 | 0.99 | 1.20 | 0.64 | 2.23 |
|  | Anaphylactic reaction | 10 | 16274 | 25305 | 11781240 | -1.75 | -2.83 | -1.03 | 0.30 | 0.16 | 0.55 |
|  | Necrotising soft tissue infection | 9 | 16275 | 18 | 11806527 | 4.14 | **3.01** | 4.91 | 17.68 | **7.94** | 39.37 |
|  | Transient ischaemic attack | 9 | 16275 | 4459 | 11802086 | 0.51 | -0.63 | 1.28 | 1.43 | 0.74 | 2.75 |
|  | Urinary retention | 9 | 16275 | 2908 | 11803637 | 1.07 | -0.07 | 1.83 | 2.10 | **1.09** | 4.05 |
|  | Drug-induced liver injury | 9 | 16275 | 9754 | 11796791 | -0.55 | -1.69 | 0.21 | 0.68 | 0.35 | 1.31 |
|  | Cellulitis of male external genital organ | 9 | 16275 | 44 | 11806501 | 4.05 | **2.91** | 4.81 | 16.58 | **8.09** | 33.97 |
|  | Breast cancer | 9 | 16275 | 33245 | 11773300 | -2.29 | -3.42 | -1.52 | 0.21 | 0.11 | 0.39 |
|  | Seizure | 8 | 16276 | 17042 | 11789503 | -1.50 | -2.71 | -0.69 | 0.35 | 0.18 | 0.71 |
|  | Hepatic cancer | 8 | 16276 | 7109 | 11799436 | -0.28 | -1.49 | 0.53 | 0.83 | 0.41 | 1.65 |
|  | Gastric cancer | 8 | 16276 | 8761 | 11797784 | -0.57 | -1.78 | 0.24 | 0.68 | 0.34 | 1.35 |
|  | Diabetic coma | 8 | 16276 | 457 | 11806088 | 2.90 | **1.69** | 3.70 | 7.45 | **3.70** | 15.00 |
|  | Gastrointestinal haemorrhage | 8 | 16276 | 43575 | 11762970 | -2.83 | -4.04 | -2.03 | 0.14 | 0.07 | 0.28 |
|  | Apparent death | 8 | 16276 | 2609 | 11803936 | 1.05 | -0.16 | 1.85 | 2.07 | **1.03** | 4.15 |
|  | Circulatory collapse | 8 | 16276 | 3990 | 11802555 | 0.50 | -0.71 | 1.30 | 1.42 | 0.71 | 2.83 |
|  | Perineal infection | 7 | 16277 | 14 | 11806531 | 3.83 | **2.52** | 4.68 | 14.18 | **5.72** | 35.14 |
|  | Pemphigoid | 7 | 16277 | 2563 | 11803982 | 0.89 | -0.41 | 1.74 | 1.86 | 0.88 | 3.90 |
|  | Interstitial lung disease | 7 | 16277 | 13085 | 11793460 | -1.31 | -2.61 | -0.45 | 0.40 | 0.19 | 0.85 |
|  | Hyperglycaemic hyperosmolar nonketotic syndrome | 7 | 16277 | 125 | 11806420 | 3.46 | **2.16** | 4.31 | 11.00 | **5.14** | 23.55 |
|  | Depressed level of consciousness | 7 | 16277 | 7079 | 11799466 | -0.45 | -1.75 | 0.40 | 0.73 | 0.35 | 1.53 |
|  | Hepatic cirrhosis | 7 | 16277 | 3208 | 11803337 | 0.61 | -0.70 | 1.46 | 1.52 | 0.72 | 3.20 |
|  | Angina unstable | 7 | 16277 | 2947 | 11803598 | 0.72 | -0.59 | 1.57 | 1.64 | 0.78 | 3.45 |
|  | Acute coronary syndrome | 7 | 16277 | 4849 | 11801696 | 0.06 | -1.24 | 0.91 | 1.04 | 0.50 | 2.19 |
|  | Cholecystitis | 7 | 16277 | 3384 | 11803161 | 0.54 | -0.77 | 1.39 | 1.45 | 0.69 | 3.05 |
|  | Bacteraemia | 7 | 16277 | 4171 | 11802374 | 0.26 | -1.04 | 1.11 | 1.20 | 0.57 | 2.52 |
|  | Brain oedema | 7 | 16277 | 3270 | 11803275 | 0.58 | -0.72 | 1.43 | 1.50 | 0.71 | 3.14 |
|  | Septic shock | 6 | 16278 | 1871 | 11804674 | 1.08 | -0.34 | 1.99 | 2.11 | 0.95 | 4.70 |
|  | Pancreatitis necrotising | 6 | 16278 | 294 | 11806251 | 2.83 | **1.42** | 3.74 | 7.12 | **3.17** | 15.98 |
|  | Neuropathy peripheral | 6 | 16278 | 12904 | 11793641 | -1.49 | -2.91 | -0.58 | 0.36 | 0.16 | 0.79 |
|  | Rheumatoid arthritis | 6 | 16278 | 7470 | 11799075 | -0.73 | -2.15 | 0.18 | 0.60 | 0.27 | 1.34 |
|  | Vulval abscess | 6 | 16278 | 46 | 11806499 | 3.51 | **2.09** | 4.42 | 11.37 | **4.86** | 26.63 |
|  | Escherichia infection | 6 | 16278 | 1103 | 11805442 | 1.68 | **0.27** | 2.59 | 3.21 | **1.44** | 7.15 |
|  | Epididymitis | 6 | 16278 | 262 | 11806283 | 2.90 | **1.49** | 3.81 | 7.48 | **3.33** | 16.80 |
|  | Lacunar infarction | 6 | 16278 | 332 | 11806213 | 2.75 | **1.34** | 3.66 | 6.73 | **3.00** | 15.10 |
|  | Femur fracture | 6 | 16278 | 5617 | 11800928 | -0.34 | -1.76 | 0.57 | 0.79 | 0.35 | 1.76 |
|  | Coronary artery occlusion | 6 | 16278 | 2775 | 11803770 | 0.59 | -0.83 | 1.50 | 1.50 | 0.67 | 3.34 |
|  | Colitis ulcerative | 6 | 16278 | 12628 | 11793917 | -1.46 | -2.88 | -0.55 | 0.36 | 0.16 | 0.81 |
|  | Coeliac disease | 6 | 16278 | 3642 | 11802903 | 0.23 | -1.18 | 1.15 | 1.18 | 0.53 | 2.62 |
|  | Blindness unilateral | 6 | 16278 | 5866 | 11800679 | -0.40 | -1.82 | 0.51 | 0.76 | 0.34 | 1.69 |
|  | Cardiac failure acute | 6 | 16278 | 2001 | 11804544 | 0.99 | -0.42 | 1.90 | 1.99 | 0.89 | 4.44 |
|  | Cerebellar infarction | 6 | 16278 | 564 | 11805981 | 2.34 | **0.92** | 3.25 | 5.06 | **2.26** | 11.31 |
|  | Cholecystitis acute | 6 | 16278 | 1664 | 11804881 | 1.21 | -0.20 | 2.13 | 2.32 | **1.04** | 5.18 |
|  | Appendicitis | 6 | 16278 | 3756 | 11802789 | 0.19 | -1.22 | 1.11 | 1.14 | 0.51 | 2.55 |
|  | Stevens-Johnson syndrome | 5 | 16279 | 3284 | 11803261 | 0.13 | -1.43 | 1.11 | 1.09 | 0.45 | 2.63 |
|  | Scrotal abscess | 5 | 16279 | 42 | 11806503 | 3.28 | **1.72** | 4.27 | 9.74 | **3.85** | 24.62 |
|  | Thrombosis | 5 | 16279 | 11596 | 11794949 | -1.58 | -3.15 | -0.60 | 0.33 | 0.14 | 0.80 |
|  | Pancreatitis relapsing | 5 | 16279 | 131 | 11806414 | 3.00 | **1.44** | 3.98 | 8.00 | **3.28** | 19.55 |
|  | Tubulointerstitial nephritis | 5 | 16279 | 1659 | 11804886 | 0.98 | -0.58 | 1.96 | 1.97 | 0.82 | 4.74 |
|  | Diverticulitis | 5 | 16279 | 7512 | 11799033 | -0.98 | -2.54 | 0.00 | 0.51 | 0.21 | 1.22 |
|  | Emphysematous pyelonephritis | 5 | 16279 | 23 | 11806522 | 3.35 | **1.79** | 4.34 | 10.21 | **3.88** | 26.87 |
|  | Liver injury | 5 | 16279 | 3179 | 11803366 | 0.17 | -1.39 | 1.16 | 1.13 | 0.47 | 2.71 |
|  | Hepatitis | 5 | 16279 | 5010 | 11801535 | -0.43 | -1.99 | 0.56 | 0.74 | 0.31 | 1.78 |
|  | Bladder cancer recurrent | 5 | 16279 | 559 | 11805986 | 2.11 | **0.54** | 3.09 | 4.31 | **1.79** | 10.39 |
|  | Deafness | 5 | 16279 | 8613 | 11797932 | -1.17 | -2.73 | -0.18 | 0.44 | 0.18 | 1.07 |
|  | Arthritis bacterial | 5 | 16279 | 1713 | 11804832 | 0.94 | -0.62 | 1.92 | 1.92 | 0.80 | 4.62 |
|  | Ascites | 5 | 16279 | 10086 | 11796459 | -1.39 | -2.95 | -0.40 | 0.38 | 0.16 | 0.92 |
|  | Acute respiratory distress syndrome | 5 | 16279 | 7138 | 11799407 | -0.91 | -2.47 | 0.07 | 0.53 | 0.22 | 1.28 |
|  | Bile duct cancer | 5 | 16279 | 426 | 11806119 | 2.33 | **0.77** | 3.31 | 5.03 | **2.08** | 12.15 |
|  | Bacterial sepsis | 5 | 16279 | 1055 | 11805490 | 1.49 | -0.07 | 2.47 | 2.81 | **1.17** | 6.76 |
|  | Systemic candida | 4 | 16280 | 103 | 11806442 | 2.80 | **1.03** | 3.88 | 6.95 | **2.56** | 18.88 |
|  | Pyelonephritis acute | 4 | 16280 | 228 | 11806317 | 2.46 | **0.69** | 3.54 | 5.49 | **2.04** | 14.76 |
|  | Thrombotic cerebral infarction | 4 | 16280 | 103 | 11806442 | 2.80 | **1.03** | 3.88 | 6.95 | **2.56** | 18.88 |
|  | Renal abscess | 4 | 16280 | 81 | 11806464 | 2.87 | **1.10** | 3.95 | 7.29 | **2.67** | 19.90 |
|  | Syncope | 4 | 16280 | 5464 | 11801081 | -0.84 | -2.60 | 0.24 | 0.56 | 0.21 | 1.49 |
|  | Renal cancer | 4 | 16280 | 16219 | 11790326 | -2.34 | -4.11 | -1.26 | 0.20 | 0.07 | 0.52 |
|  | Thrombocytopenia | 4 | 16280 | 6932 | 11799613 | -1.16 | -2.92 | -0.08 | 0.45 | 0.17 | 1.19 |
|  | Myocardial ischaemia | 4 | 16280 | 1758 | 11804787 | 0.62 | -1.14 | 1.70 | 1.54 | 0.58 | 4.10 |
|  | Prostate cancer | 4 | 16280 | 21866 | 11784679 | -2.77 | -4.53 | -1.69 | 0.15 | 0.06 | 0.39 |
|  | Ventricular tachycardia | 4 | 16280 | 734 | 11805811 | 1.57 | -0.20 | 2.65 | 2.97 | **1.11** | 7.93 |
|  | Epilepsy | 4 | 16280 | 7409 | 11799136 | -1.25 | -3.02 | -0.17 | 0.42 | 0.16 | 1.12 |
|  | Haemorrhage | 4 | 16280 | 24059 | 11782486 | -2.90 | -4.67 | -1.82 | 0.13 | 0.05 | 0.36 |
|  | Diabetic metabolic decompensation | 4 | 16280 | 537 | 11806008 | 1.85 | **0.09** | 2.93 | 3.61 | **1.35** | 9.67 |
|  | Infected skin ulcer | 4 | 16280 | 355 | 11806190 | 2.18 | **0.41** | 3.26 | 4.53 | **1.69** | 12.12 |
|  | Emphysematous cystitis | 4 | 16280 | 32 | 11806513 | 3.03 | **1.27** | 4.11 | 8.19 | **2.90** | 23.16 |
|  | Hepatic failure | 4 | 16280 | 5427 | 11801118 | -0.83 | -2.59 | 0.25 | 0.56 | 0.21 | 1.50 |
|  | Diabetic ketoacidotic hyperglycaemic coma | 4 | 16280 | 46 | 11806499 | 2.98 | **1.22** | 4.06 | 7.91 | **2.85** | 21.98 |
|  | Hepatocellular injury | 4 | 16280 | 4627 | 11801918 | -0.61 | -2.38 | 0.47 | 0.65 | 0.25 | 1.74 |
|  | Anuria | 4 | 16280 | 2484 | 11804061 | 0.20 | -1.57 | 1.28 | 1.15 | 0.43 | 3.06 |
|  | Coronary artery stenosis | 4 | 16280 | 560 | 11805985 | 1.82 | **0.05** | 2.90 | 3.52 | **1.32** | 9.42 |
|  | Dementia Alzheimers type | 4 | 16280 | 2739 | 11803806 | 0.07 | -1.69 | 1.15 | 1.05 | 0.39 | 2.81 |
|  | Cerebral haematoma | 4 | 16280 | 1465 | 11805080 | 0.83 | -0.93 | 1.91 | 1.78 | 0.67 | 4.76 |
|  | Deafness unilateral | 4 | 16280 | 1640 | 11804905 | 0.70 | -1.06 | 1.78 | 1.63 | 0.61 | 4.34 |
|  | Abdominal abscess | 4 | 16280 | 2773 | 11803772 | 0.06 | -1.71 | 1.14 | 1.04 | 0.39 | 2.77 |
|  | Penile cancer | 3 | 16281 | 60 | 11806485 | 2.58 | **0.51** | 3.78 | 5.96 | **1.87** | 19.02 |
|  | Pyonephrosis | 3 | 16281 | 1 | 11806544 | 2.79 | **0.72** | 4.00 | 6.92 | 0.72 | 66.57 |
|  | Thyroid cancer | 3 | 16281 | 3304 | 11803241 | -0.53 | -2.60 | 0.68 | 0.69 | 0.22 | 2.15 |
|  | Renal injury | 3 | 16281 | 10395 | 11796150 | -2.08 | -4.15 | -0.88 | 0.24 | 0.08 | 0.73 |
|  | Nephrotic syndrome | 3 | 16281 | 1068 | 11805477 | 0.83 | -1.24 | 2.03 | 1.77 | 0.57 | 5.50 |
|  | Nephritis | 3 | 16281 | 518 | 11806027 | 1.52 | -0.55 | 2.73 | 2.87 | 0.92 | 8.94 |
|  | Rhabdomyolysis | 3 | 16281 | 3337 | 11803208 | -0.54 | -2.61 | 0.66 | 0.69 | 0.22 | 2.13 |
|  | Pulmonary oedema | 3 | 16281 | 2996 | 11803549 | -0.40 | -2.47 | 0.80 | 0.76 | 0.24 | 2.35 |
|  | Retinal artery occlusion | 3 | 16281 | 363 | 11806182 | 1.80 | -0.27 | 3.01 | 3.49 | **1.12** | 10.86 |
|  | Oesophageal candidiasis | 3 | 16281 | 486 | 11806059 | 1.58 | -0.49 | 2.78 | 2.98 | 0.96 | 9.28 |
|  | Eye haemorrhage | 3 | 16281 | 3038 | 11803507 | -0.42 | -2.49 | 0.78 | 0.75 | 0.24 | 2.32 |
|  | Empyema | 3 | 16281 | 382 | 11806163 | 1.76 | -0.31 | 2.97 | 3.40 | **1.09** | 10.58 |
|  | Malignant melanoma | 3 | 16281 | 6244 | 11800301 | -1.38 | -3.45 | -0.17 | 0.38 | 0.12 | 1.19 |
|  | Hip fracture | 3 | 16281 | 4094 | 11802451 | -0.81 | -2.88 | 0.39 | 0.57 | 0.18 | 1.77 |
|  | Lip and/or oral cavity cancer | 3 | 16281 | 395 | 11806150 | 1.74 | -0.33 | 2.95 | 3.34 | **1.07** | 10.40 |
|  | Haematochezia | 3 | 16281 | 5439 | 11801106 | -1.19 | -3.26 | 0.01 | 0.44 | 0.14 | 1.36 |
|  | Hepatotoxicity | 3 | 16281 | 4167 | 11802378 | -0.83 | -2.90 | 0.37 | 0.56 | 0.18 | 1.74 |
|  | Escherichia sepsis | 3 | 16281 | 567 | 11805978 | 1.45 | -0.62 | 2.65 | 2.72 | 0.88 | 8.47 |
|  | Diabetes insipidus | 3 | 16281 | 668 | 11805877 | 1.30 | -0.77 | 2.50 | 2.46 | 0.79 | 7.64 |
|  | Glaucoma | 3 | 16281 | 3568 | 11802977 | -0.63 | -2.70 | 0.58 | 0.65 | 0.21 | 2.00 |
|  | Hallucination | 3 | 16281 | 9196 | 11797349 | -1.91 | -3.98 | -0.71 | 0.27 | 0.09 | 0.82 |
|  | Alcoholic ketoacidosis | 3 | 16281 | 1 | 11806544 | 2.79 | **0.72** | 4.00 | 6.92 | 0.72 | 66.57 |
|  | Autoimmune disorder | 3 | 16281 | 2616 | 11803929 | -0.23 | -2.30 | 0.98 | 0.85 | 0.27 | 2.64 |
|  | Cholestasis | 3 | 16281 | 6119 | 11800426 | -1.35 | -3.42 | -0.15 | 0.39 | 0.13 | 1.22 |
|  | Basedows disease | 3 | 16281 | 1180 | 11805365 | 0.72 | -1.35 | 1.92 | 1.64 | 0.53 | 5.10 |
|  | Dementia | 3 | 16281 | 7737 | 11798808 | -1.67 | -3.74 | -0.47 | 0.31 | 0.10 | 0.97 |
|  | Anaphylactic shock | 3 | 16281 | 10842 | 11795703 | -2.14 | -4.21 | -0.93 | 0.23 | 0.07 | 0.70 |
|  | Cerebral haemorrhage | 3 | 16281 | 17072 | 11789473 | -2.78 | -4.85 | -1.57 | 0.15 | 0.05 | 0.45 |
|  | Brain stem infarction | 3 | 16281 | 411 | 11806134 | 1.71 | -0.36 | 2.92 | 3.27 | **1.05** | 10.18 |
|  | Colitis ischaemic | 3 | 16281 | 2227 | 11804318 | -0.03 | -2.10 | 1.18 | 0.98 | 0.32 | 3.04 |
|  | Carotid artery stenosis | 3 | 16281 | 591 | 11805954 | 1.41 | -0.66 | 2.62 | 2.66 | 0.85 | 8.26 |
|  | Coma scale abnormal | 3 | 16281 | 764 | 11805781 | 1.17 | -0.90 | 2.38 | 2.25 | 0.72 | 6.99 |
|  | Aortic stenosis | 3 | 16281 | 1338 | 11805207 | 0.58 | -1.49 | 1.78 | 1.49 | 0.48 | 4.63 |
|  | COVID-19 pneumonia | 3 | 16281 | 2186 | 11804359 | -0.01 | -2.08 | 1.20 | 1.00 | 0.32 | 3.09 |
| Dapagliflozin | Diabetic ketoacidosis | 1438 | 10854 | 8570 | 11801967 | 7.04 | **6.96** | 7.11 | 131.91 | **124.36** | 139.92 |
|  | Ketoacidosis | 384 | 11908 | 1716 | 11808821 | 7.16 | **6.99** | 7.29 | 143.29 | **128.10** | 160.29 |
|  | Acute kidney injury | 244 | 12048 | 115873 | 11694664 | 1.01 | **0.80** | 1.17 | 2.02 | **1.78** | 2.29 |
|  | Euglycaemic diabetic ketoacidosis | 240 | 12052 | 863 | 11809674 | 7.19 | **6.98** | 7.34 | 146.04 | **126.44** | 168.69 |
|  | Death | 220 | 12072 | 418558 | 11391979 | -0.98 | -1.21 | -0.82 | 0.51 | 0.44 | 0.58 |
|  | Cerebral infarction | 103 | 12189 | 9800 | 11800737 | 3.26 | **2.93** | 3.50 | 9.59 | **7.89** | 11.65 |
|  | Fournier’s gangrene | 102 | 12190 | 784 | 11809753 | 6.17 | **5.84** | 6.41 | 72.12 | **58.63** | 88.72 |
|  | Bladder cancer | 89 | 12203 | 28520 | 11782017 | 1.57 | **1.21** | 1.82 | 2.96 | **2.40** | 3.65 |
|  | Diabetes mellitus inadequate control | 85 | 12207 | 4271 | 11806266 | 4.09 | **3.73** | 4.35 | 17.00 | **13.71** | 21.09 |
|  | Pancreatitis | 70 | 12222 | 10687 | 11799850 | 2.59 | **2.20** | 2.88 | 6.03 | **4.77** | 7.64 |
|  | Cerebrovascular accident | 61 | 12231 | 67490 | 11743047 | -0.20 | -0.63 | 0.10 | 0.87 | 0.68 | 1.12 |
|  | Myocardial infarction | 58 | 12234 | 48311 | 11762226 | 0.20 | -0.23 | 0.52 | 1.15 | 0.89 | 1.49 |
|  | Necrotising fasciitis | 45 | 12247 | 620 | 11809917 | 5.26 | **4.76** | 5.61 | 38.19 | **28.20** | 51.71 |
|  | Acute myocardial infarction | 44 | 12248 | 15541 | 11794996 | 1.41 | **0.91** | 1.77 | 2.66 | **1.98** | 3.58 |
|  | Atrial fibrillation | 40 | 12252 | 39556 | 11770981 | -0.04 | -0.57 | 0.33 | 0.97 | 0.71 | 1.33 |
|  | Cardiac failure | 38 | 12254 | 27814 | 11782723 | 0.39 | -0.15 | 0.77 | 1.31 | 0.95 | 1.80 |
|  | Cellulitis | 38 | 12254 | 18983 | 11791554 | 0.93 | **0.38** | 1.31 | 1.90 | **1.38** | 2.61 |
|  | Renal impairment | 37 | 12255 | 8493 | 11802044 | 2.00 | **1.45** | 2.39 | 4.00 | **2.90** | 5.53 |
|  | Renal failure | 30 | 12262 | 12550 | 11797987 | 1.17 | **0.56** | 1.60 | 2.25 | **1.57** | 3.22 |
|  | Pancreatitis acute | 28 | 12264 | 5078 | 11805459 | 2.29 | **1.66** | 2.74 | 4.91 | **3.38** | 7.12 |
|  | Angina pectoris | 28 | 12264 | 12880 | 11797657 | 1.03 | **0.40** | 1.48 | 2.05 | **1.41** | 2.97 |
|  | Sepsis | 27 | 12265 | 8375 | 11802162 | 1.57 | **0.93** | 2.03 | 2.98 | **2.04** | 4.35 |
|  | Diabetic ketosis | 26 | 12266 | 75 | 11810462 | 5.45 | **4.80** | 5.92 | 43.80 | **28.03** | 68.45 |
|  | Urosepsis | 25 | 12267 | 696 | 11809841 | 4.35 | **3.68** | 4.82 | 20.41 | **13.69** | 30.42 |
|  | Hyperkalaemia | 25 | 12267 | 4062 | 11806475 | 2.42 | **1.76** | 2.90 | 5.37 | **3.62** | 7.96 |
|  | Arrhythmia | 23 | 12269 | 19446 | 11791091 | 0.18 | -0.52 | 0.67 | 1.13 | 0.75 | 1.71 |
|  | Gangrene | 21 | 12271 | 1263 | 11809274 | 3.55 | **2.82** | 4.06 | 11.72 | **7.61** | 18.04 |
|  | Syncope | 20 | 12272 | 5448 | 11805089 | 1.73 | **0.98** | 2.25 | 3.31 | **2.14** | 5.14 |
|  | Nephritis | 20 | 12272 | 501 | 11810036 | 4.30 | **3.55** | 4.82 | 19.68 | **12.58** | 30.78 |
|  | Altered state of consciousness | 20 | 12272 | 9613 | 11800924 | 0.96 | **0.21** | 1.49 | 1.95 | **1.26** | 3.02 |
|  | Nephrolithiasis | 18 | 12274 | 6796 | 11803741 | 1.29 | **0.49** | 1.84 | 2.44 | **1.54** | 3.88 |
|  | Angioedema | 18 | 12274 | 21511 | 11789026 | -0.31 | -1.10 | 0.24 | 0.81 | 0.51 | 1.28 |
|  | Pyelonephritis | 17 | 12275 | 1005 | 11809532 | 3.49 | **2.67** | 4.05 | 11.20 | **6.93** | 18.09 |
|  | Lactic acidosis | 17 | 12275 | 3163 | 11807374 | 2.20 | **1.38** | 2.77 | 4.60 | **2.85** | 7.41 |
|  | Erectile dysfunction | 17 | 12275 | 4595 | 11805942 | 1.72 | **0.91** | 2.29 | 3.30 | **2.05** | 5.32 |
|  | Diabetes mellitus | 17 | 12275 | 17117 | 11793420 | -0.07 | -0.88 | 0.50 | 0.96 | 0.59 | 1.54 |
|  | Angina unstable | 17 | 12275 | 2937 | 11807600 | 2.29 | **1.48** | 2.86 | 4.90 | **3.04** | 7.90 |
|  | Deep vein thrombosis | 17 | 12275 | 23639 | 11786898 | -0.52 | -1.34 | 0.05 | 0.70 | 0.43 | 1.12 |
|  | Pancreatic carcinoma | 16 | 12276 | 9936 | 11800601 | 0.61 | -0.24 | 1.19 | 1.52 | 0.93 | 2.48 |
|  | Cystitis haemorrhagic | 16 | 12276 | 1209 | 11809328 | 3.22 | **2.38** | 3.80 | 9.30 | **5.68** | 15.24 |
|  | Pneumonia | 15 | 12277 | 49774 | 11760763 | -1.75 | -2.62 | -1.15 | 0.30 | 0.18 | 0.49 |
|  | Prostate cancer | 15 | 12277 | 21855 | 11788682 | -0.58 | -1.46 | 0.02 | 0.67 | 0.40 | 1.11 |
|  | Colon cancer | 15 | 12277 | 5987 | 11804550 | 1.20 | **0.33** | 1.80 | 2.30 | **1.39** | 3.82 |
|  | Breast cancer female | 15 | 12277 | 3021 | 11807516 | 2.08 | **1.21** | 2.69 | 4.24 | **2.55** | 7.04 |
|  | Renal cancer | 14 | 12278 | 16209 | 11794328 | -0.26 | -1.16 | 0.36 | 0.83 | 0.49 | 1.41 |
|  | Haemorrhage | 14 | 12278 | 24049 | 11786488 | -0.82 | -1.72 | -0.19 | 0.57 | 0.34 | 0.96 |
|  | Coma | 14 | 12278 | 12232 | 11798305 | 0.13 | -0.77 | 0.75 | 1.10 | 0.65 | 1.85 |
|  | Urinary retention | 13 | 12279 | 2904 | 11807633 | 1.93 | **1.00** | 2.58 | 3.82 | **2.22** | 6.59 |
|  | Transient ischaemic attack | 13 | 12279 | 4455 | 11806082 | 1.39 | **0.45** | 2.03 | 2.62 | **1.52** | 4.52 |
|  | Epididymitis | 12 | 12280 | 256 | 11810281 | 4.00 | **3.03** | 4.67 | 16.05 | **9.00** | 28.65 |
|  | Cardiac failure congestive | 12 | 12280 | 33873 | 11776664 | -1.52 | -2.49 | -0.85 | 0.35 | 0.20 | 0.62 |
|  | Brain stem infarction | 12 | 12280 | 402 | 11810135 | 3.75 | **2.77** | 4.42 | 13.43 | **7.56** | 23.86 |
|  | Blindness | 11 | 12281 | 16981 | 11793556 | -0.66 | -1.68 | 0.04 | 0.63 | 0.35 | 1.14 |
|  | Anaphylactic reaction | 11 | 12281 | 25304 | 11785233 | -1.22 | -2.25 | -0.53 | 0.43 | 0.24 | 0.77 |
|  | Chronic kidney disease | 10 | 12282 | 52179 | 11758358 | -2.38 | -3.46 | -1.66 | 0.19 | 0.10 | 0.36 |
|  | Cardiac arrest | 10 | 12282 | 26788 | 11783749 | -1.43 | -2.51 | -0.71 | 0.37 | 0.20 | 0.69 |
|  | Pyelonephritis acute | 9 | 12283 | 223 | 11810314 | 3.68 | **2.54** | 4.44 | 12.82 | **6.58** | 24.96 |
|  | Necrosis | 9 | 12283 | 465 | 11810072 | 3.26 | **2.12** | 4.02 | 9.57 | **4.95** | 18.51 |
|  | Drug-induced liver injury | 9 | 12283 | 9754 | 11800783 | -0.16 | -1.30 | 0.60 | 0.89 | 0.46 | 1.72 |
|  | Hypoglycaemic coma | 9 | 12283 | 702 | 11809835 | 2.94 | **1.80** | 3.70 | 7.67 | **3.97** | 14.80 |
|  | Diverticulitis | 9 | 12283 | 7508 | 11803029 | 0.19 | -0.95 | 0.95 | 1.14 | 0.59 | 2.20 |
|  | Ischaemic stroke | 9 | 12283 | 5457 | 11805080 | 0.62 | -0.52 | 1.38 | 1.54 | 0.80 | 2.96 |
|  | Appendicitis | 9 | 12283 | 3753 | 11806784 | 1.11 | -0.03 | 1.87 | 2.15 | **1.12** | 4.14 |
|  | Breast cancer | 9 | 12283 | 33245 | 11777292 | -1.88 | -3.02 | -1.12 | 0.27 | 0.14 | 0.52 |
|  | Cataract | 9 | 12283 | 20096 | 11790441 | -1.17 | -2.31 | -0.41 | 0.44 | 0.23 | 0.85 |
|  | Cardiac failure acute | 9 | 12283 | 1998 | 11808539 | 1.88 | **0.74** | 2.64 | 3.67 | **1.91** | 7.07 |
|  | Bladder transitional cell carcinoma | 9 | 12283 | 891 | 11809646 | 2.73 | **1.59** | 3.49 | 6.62 | **3.43** | 12.76 |
|  | Pemphigoid | 8 | 12284 | 2562 | 11807975 | 1.42 | **0.21** | 2.23 | 2.68 | **1.34** | 5.37 |
|  | Seizure | 8 | 12284 | 17042 | 11793495 | -1.10 | -2.31 | -0.30 | 0.47 | 0.23 | 0.93 |
|  | Anal abscess | 8 | 12284 | 2706 | 11807831 | 1.36 | **0.14** | 2.16 | 2.56 | **1.28** | 5.12 |
|  | Asphyxia | 8 | 12284 | 3472 | 11807065 | 1.05 | -0.17 | 1.85 | 2.06 | **1.03** | 4.13 |
|  | Thrombotic cerebral infarction | 7 | 12285 | 100 | 11810437 | 3.62 | **2.31** | 4.47 | 12.27 | **5.70** | 26.41 |
|  | Neoplasm malignant | 7 | 12285 | 16348 | 11794189 | -1.22 | -2.53 | -0.37 | 0.43 | 0.20 | 0.90 |
|  | Thrombocytopenia | 7 | 12285 | 6929 | 11803608 | -0.04 | -1.34 | 0.81 | 0.97 | 0.46 | 2.04 |
|  | Disseminated intravascular coagulation | 7 | 12285 | 2578 | 11807959 | 1.23 | -0.07 | 2.09 | 2.35 | **1.12** | 4.94 |
|  | Depressed level of consciousness | 7 | 12285 | 7079 | 11803458 | -0.07 | -1.37 | 0.78 | 0.95 | 0.45 | 2.00 |
|  | Lung neoplasm malignant | 7 | 12285 | 10905 | 11799632 | -0.66 | -1.96 | 0.19 | 0.63 | 0.30 | 1.33 |
|  | Loss of consciousness | 7 | 12285 | 8847 | 11801690 | -0.37 | -1.67 | 0.48 | 0.77 | 0.37 | 1.62 |
|  | Cellulitis of male external genital organ | 7 | 12285 | 46 | 11810491 | 3.76 | **2.45** | 4.61 | 13.51 | **6.10** | 29.93 |
|  | Acute coronary syndrome | 7 | 12285 | 4849 | 11805688 | 0.43 | -0.87 | 1.29 | 1.35 | 0.64 | 2.84 |
|  | Coronary artery occlusion | 7 | 12285 | 2774 | 11807763 | 1.15 | -0.16 | 2.00 | 2.21 | **1.05** | 4.64 |
|  | Pulmonary embolism | 6 | 12286 | 14667 | 11795870 | -1.28 | -2.69 | -0.37 | 0.41 | 0.19 | 0.92 |
|  | Transitional cell carcinoma | 6 | 12286 | 271 | 11810266 | 3.04 | **1.63** | 3.96 | 8.25 | **3.67** | 18.53 |
|  | Thrombosis | 6 | 12286 | 11595 | 11798942 | -0.95 | -2.37 | -0.04 | 0.52 | 0.23 | 1.15 |
|  | Rhabdomyolysis | 6 | 12286 | 3334 | 11807203 | 0.71 | -0.70 | 1.62 | 1.64 | 0.73 | 3.65 |
|  | Escherichia bacteraemia | 6 | 12286 | 307 | 11810230 | 2.98 | **1.56** | 3.89 | 7.87 | **3.51** | 17.67 |
|  | Ileus | 6 | 12286 | 2211 | 11808326 | 1.21 | -0.20 | 2.12 | 2.32 | **1.04** | 5.16 |
|  | Hypokalaemia | 6 | 12286 | 4337 | 11806200 | 0.37 | -1.04 | 1.29 | 1.30 | 0.58 | 2.89 |
|  | Lacunar infarction | 6 | 12286 | 332 | 11810205 | 2.93 | **1.52** | 3.84 | 7.63 | **3.40** | 17.12 |
|  | Hyperglycaemic hyperosmolar nonketotic syndrome | 6 | 12286 | 126 | 11810411 | 3.35 | **1.94** | 4.26 | 10.20 | **4.50** | 23.14 |
|  | Hydronephrosis | 6 | 12286 | 755 | 11809782 | 2.33 | **0.92** | 3.24 | 5.03 | **2.25** | 11.24 |
|  | Cerebral haemorrhage | 6 | 12286 | 17069 | 11793468 | -1.49 | -2.90 | -0.58 | 0.36 | 0.16 | 0.79 |
|  | Bradycardia | 6 | 12286 | 16955 | 11793582 | -1.48 | -2.90 | -0.57 | 0.36 | 0.16 | 0.80 |
|  | Neuropathy peripheral | 5 | 12287 | 12905 | 11797632 | -1.34 | -2.90 | -0.36 | 0.40 | 0.16 | 0.95 |
|  | Kidney infection | 5 | 12287 | 3430 | 11807107 | 0.43 | -1.13 | 1.42 | 1.35 | 0.56 | 3.25 |
|  | Diabetic metabolic decompensation | 5 | 12287 | 536 | 11810001 | 2.37 | **0.81** | 3.36 | 5.18 | **2.15** | 12.49 |
|  | Diabetic coma | 5 | 12287 | 460 | 11810077 | 2.48 | **0.92** | 3.47 | 5.59 | **2.32** | 13.50 |
|  | Hepatitis | 5 | 12287 | 5010 | 11805527 | -0.06 | -1.62 | 0.93 | 0.96 | 0.40 | 2.31 |
|  | Diabetic hyperosmolar coma | 5 | 12287 | 95 | 11810442 | 3.19 | **1.62** | 4.17 | 9.11 | **3.70** | 22.39 |
|  | Anuria | 5 | 12287 | 2483 | 11808054 | 0.83 | -0.73 | 1.82 | 1.78 | 0.74 | 4.29 |
|  | Colitis ischaemic | 5 | 12287 | 2225 | 11808312 | 0.96 | -0.60 | 1.95 | 1.95 | 0.81 | 4.69 |
|  | COVID-19 pneumonia | 5 | 12287 | 2184 | 11808353 | 0.99 | -0.58 | 1.97 | 1.98 | 0.82 | 4.77 |
|  | Acute respiratory failure | 5 | 12287 | 8421 | 11802116 | -0.75 | -2.31 | 0.23 | 0.59 | 0.25 | 1.43 |
|  | Apparent death | 5 | 12287 | 2612 | 11807925 | 0.77 | -0.79 | 1.76 | 1.71 | 0.71 | 4.11 |
|  | Sudden death | 4 | 12288 | 2660 | 11807877 | 0.46 | -1.30 | 1.54 | 1.38 | 0.52 | 3.67 |
|  | Near death experience | 4 | 12288 | 185 | 11810352 | 2.69 | **0.93** | 3.77 | 6.46 | **2.40** | 17.40 |
|  | Pancreatitis relapsing | 4 | 12288 | 132 | 11810405 | 2.81 | **1.05** | 3.89 | 7.02 | **2.59** | 18.97 |
|  | Tubulointerstitial nephritis | 4 | 12288 | 1660 | 11808877 | 1.01 | -0.75 | 2.09 | 2.02 | 0.76 | 5.38 |
|  | Renal abscess | 4 | 12288 | 81 | 11810456 | 2.94 | **1.17** | 4.01 | 7.65 | **2.80** | 20.87 |
|  | Myocardial ischaemia | 4 | 12288 | 1758 | 11808779 | 0.95 | -0.82 | 2.03 | 1.93 | 0.72 | 5.15 |
|  | Scrotal abscess | 4 | 12288 | 43 | 11810494 | 3.04 | **1.27** | 4.11 | 8.20 | **2.94** | 22.84 |
|  | Ventricular tachycardia | 4 | 12288 | 734 | 11809803 | 1.83 | **0.06** | 2.91 | 3.55 | **1.33** | 9.49 |
|  | Rectal haemorrhage | 4 | 12288 | 4727 | 11805810 | -0.27 | -2.03 | 0.81 | 0.83 | 0.31 | 2.21 |
|  | Epilepsy | 4 | 12288 | 7409 | 11803128 | -0.87 | -2.63 | 0.21 | 0.55 | 0.21 | 1.46 |
|  | Interstitial lung disease | 4 | 12288 | 13088 | 11797449 | -1.65 | -3.41 | -0.57 | 0.32 | 0.12 | 0.85 |
|  | Erythema multiforme | 4 | 12288 | 2826 | 11807711 | 0.39 | -1.38 | 1.47 | 1.31 | 0.49 | 3.49 |
|  | Escherichia urinary tract infection | 4 | 12288 | 700 | 11809837 | 1.87 | **0.10** | 2.95 | 3.65 | **1.37** | 9.76 |
|  | Escherichia sepsis | 4 | 12288 | 566 | 11809971 | 2.04 | **0.28** | 3.12 | 4.12 | **1.54** | 11.01 |
|  | Hip fracture | 4 | 12288 | 4093 | 11806444 | -0.08 | -1.85 | 1.00 | 0.95 | 0.35 | 2.52 |
|  | Diabetic ketoacidotic hyperglycaemic coma | 4 | 12288 | 46 | 11810491 | 3.03 | **1.26** | 4.11 | 8.15 | **2.93** | 22.65 |
|  | Hallucination | 4 | 12288 | 9195 | 11801342 | -1.16 | -2.93 | -0.08 | 0.45 | 0.17 | 1.19 |
|  | Hypertensive crisis | 4 | 12288 | 2154 | 11808383 | 0.71 | -1.05 | 1.79 | 1.64 | 0.61 | 4.37 |
|  | Gastric cancer | 4 | 12288 | 8765 | 11801772 | -1.10 | -2.86 | -0.02 | 0.47 | 0.18 | 1.25 |
|  | Gastrointestinal haemorrhage | 4 | 12288 | 43579 | 11766958 | -3.35 | -5.11 | -2.27 | 0.10 | 0.04 | 0.26 |
|  | Delirium | 4 | 12288 | 7194 | 11803343 | -0.83 | -2.59 | 0.25 | 0.56 | 0.21 | 1.50 |
|  | Cholecystitis | 4 | 12288 | 3387 | 11807150 | 0.16 | -1.60 | 1.24 | 1.12 | 0.42 | 2.98 |
|  | Cholangitis | 4 | 12288 | 1901 | 11808636 | 0.86 | -0.91 | 1.94 | 1.81 | 0.68 | 4.84 |
|  | Bile duct cancer | 4 | 12288 | 427 | 11810110 | 2.25 | **0.48** | 3.33 | 4.75 | **1.77** | 12.71 |
|  | Coronary artery stenosis | 4 | 12288 | 560 | 11809977 | 2.05 | **0.29** | 3.13 | 4.14 | **1.55** | 11.08 |
|  | Bladder cancer recurrent | 4 | 12288 | 560 | 11809977 | 2.05 | **0.29** | 3.13 | 4.14 | **1.55** | 11.08 |
|  | Cardio-respiratory arrest | 4 | 12288 | 14745 | 11795792 | -1.82 | -3.58 | -0.74 | 0.28 | 0.11 | 0.76 |
|  | Suicidal ideation | 3 | 12289 | 5227 | 11805310 | -0.76 | -2.83 | 0.44 | 0.59 | 0.19 | 1.83 |
|  | Oesophageal carcinoma | 3 | 12289 | 8350 | 11802187 | -1.39 | -3.46 | -0.19 | 0.38 | 0.12 | 1.18 |
|  | Renal haemorrhage | 3 | 12289 | 277 | 11810260 | 2.15 | **0.08** | 3.35 | 4.42 | **1.42** | 13.80 |
|  | Osteomyelitis | 3 | 12289 | 2288 | 11808249 | 0.28 | -1.79 | 1.49 | 1.21 | 0.39 | 3.77 |
|  | Staphylococcal infection | 3 | 12289 | 2360 | 11808177 | 0.24 | -1.83 | 1.45 | 1.18 | 0.38 | 3.67 |
|  | Ovarian cancer | 3 | 12289 | 2679 | 11807858 | 0.09 | -1.98 | 1.30 | 1.06 | 0.34 | 3.30 |
|  | Type 2 diabetes mellitus | 3 | 12289 | 12503 | 11798034 | -1.95 | -4.02 | -0.74 | 0.26 | 0.08 | 0.80 |
|  | Prostatic abscess | 3 | 12289 | 16 | 11810521 | 2.75 | **0.68** | 3.96 | 6.73 | **1.96** | 23.11 |
|  | Nephrotic syndrome | 3 | 12289 | 1068 | 11809469 | 1.12 | -0.95 | 2.32 | 2.17 | 0.70 | 6.74 |
|  | Dermatitis bullous | 3 | 12289 | 2607 | 11807930 | 0.12 | -1.95 | 1.33 | 1.09 | 0.35 | 3.38 |
|  | Liposarcoma | 3 | 12289 | 105 | 11810432 | 2.52 | **0.45** | 3.72 | 5.72 | **1.81** | 18.01 |
|  | Femoral neck fracture | 3 | 12289 | 879 | 11809658 | 1.30 | -0.76 | 2.51 | 2.47 | 0.79 | 7.67 |
|  | Enterocolitis | 3 | 12289 | 1453 | 11809084 | 0.80 | -1.27 | 2.00 | 1.74 | 0.56 | 5.40 |
|  | Haematochezia | 3 | 12289 | 5439 | 11805098 | -0.82 | -2.88 | 0.39 | 0.57 | 0.18 | 1.76 |
|  | Haemorrhagic transformation stroke | 3 | 12289 | 597 | 11809940 | 1.64 | -0.43 | 2.85 | 3.11 | **1.00** | 9.69 |
|  | Gastric ulcer haemorrhage | 3 | 12289 | 1632 | 11808905 | 0.67 | -1.40 | 1.88 | 1.59 | 0.51 | 4.94 |
|  | Diverticulum intestinal haemorrhagic | 3 | 12289 | 1365 | 11809172 | 0.86 | -1.20 | 2.07 | 1.82 | 0.59 | 5.65 |
|  | Generalised tonic-clonic seizure | 3 | 12289 | 3725 | 11806812 | -0.32 | -2.39 | 0.88 | 0.80 | 0.26 | 2.48 |
|  | Infarction | 3 | 12289 | 1757 | 11808780 | 0.59 | -1.48 | 1.79 | 1.50 | 0.48 | 4.66 |
|  | Hypersensitivity vasculitis | 3 | 12289 | 1582 | 11808955 | 0.70 | -1.36 | 1.91 | 1.63 | 0.52 | 5.06 |
|  | Disability | 3 | 12289 | 5561 | 11804976 | -0.84 | -2.91 | 0.36 | 0.56 | 0.18 | 1.73 |
|  | Gastric ulcer | 3 | 12289 | 3389 | 11807148 | -0.20 | -2.27 | 1.00 | 0.87 | 0.28 | 2.70 |
|  | Groin abscess | 3 | 12289 | 197 | 11810340 | 2.31 | **0.24** | 3.51 | 4.94 | **1.58** | 15.46 |
|  | Cholecystitis infective | 3 | 12289 | 756 | 11809781 | 1.44 | -0.63 | 2.65 | 2.72 | 0.87 | 8.44 |
|  | Anaphylactic shock | 3 | 12289 | 10842 | 11799695 | -1.75 | -3.82 | -0.54 | 0.30 | 0.10 | 0.92 |
|  | Carotid artery stenosis | 3 | 12289 | 591 | 11809946 | 1.65 | -0.42 | 2.85 | 3.13 | **1.01** | 9.74 |
|  | Atrioventricular block complete | 3 | 12289 | 2561 | 11807976 | 0.14 | -1.92 | 1.35 | 1.11 | 0.36 | 3.43 |
|  | Autoimmune hepatitis | 3 | 12289 | 2952 | 11807585 | -0.03 | -2.10 | 1.18 | 0.98 | 0.32 | 3.04 |
|  | Bladder papilloma | 3 | 12289 | 34 | 11810503 | 2.70 | **0.63** | 3.91 | 6.50 | **2.00** | 21.17 |
|  | Aortic valve incompetence | 3 | 12289 | 934 | 11809603 | 1.25 | -0.82 | 2.45 | 2.37 | 0.76 | 7.38 |
|  | Blindness unilateral | 3 | 12289 | 5869 | 11804668 | -0.92 | -2.99 | 0.29 | 0.53 | 0.17 | 1.64 |
|  | Cerebellar infarction | 3 | 12289 | 567 | 11809970 | 1.68 | -0.39 | 2.89 | 3.20 | **1.03** | 9.96 |
|  | Abdominal abscess | 3 | 12289 | 2774 | 11807763 | 0.05 | -2.02 | 1.25 | 1.03 | 0.33 | 3.21 |
|  | Acute hepatic failure | 3 | 12289 | 6943 | 11803594 | -1.14 | -3.21 | 0.06 | 0.45 | 0.15 | 1.41 |
|  | Akinesia | 3 | 12289 | 974 | 11809563 | 1.21 | -0.86 | 2.41 | 2.31 | 0.74 | 7.17 |
|  | Cholecystitis acute | 3 | 12289 | 1667 | 11808870 | 0.65 | -1.42 | 1.85 | 1.57 | 0.50 | 4.86 |
|  | Bronchiectasis | 3 | 12289 | 1756 | 11808781 | 0.59 | -1.48 | 1.79 | 1.50 | 0.48 | 4.67 |
|  | Colorectal cancer | 3 | 12289 | 22303 | 11788234 | -2.76 | -4.83 | -1.55 | 0.15 | 0.05 | 0.46 |
|  | Ascites | 3 | 12289 | 10088 | 11800449 | -1.65 | -3.72 | -0.44 | 0.32 | 0.10 | 0.99 |
|  | Autoimmune disorder | 3 | 12289 | 2616 | 11807921 | 0.12 | -1.95 | 1.33 | 1.09 | 0.35 | 3.37 |
|  | Acute febrile neutrophilic dermatosis | 3 | 12289 | 1103 | 11809434 | 1.08 | -0.98 | 2.29 | 2.12 | 0.68 | 6.59 |
| Ertugliflozin | Diabetic ketoacidosis | 20 | 492 | 9988 | 11812329 | 4.46 | **3.71** | 4.98 | 21.96 | **14.04** | 34.36 |
|  | Acute kidney injury | 8 | 504 | 116109 | 11706208 | 0.62 | -0.59 | 1.42 | 1.54 | 0.76 | 3.09 |
|  | Ketoacidosis | 5 | 507 | 2095 | 11820222 | 3.22 | **1.66** | 4.20 | 9.31 | **3.85** | 22.48 |
|  | Euglycaemic diabetic ketoacidosis | 5 | 507 | 1098 | 11821219 | 3.33 | **1.77** | 4.31 | 10.04 | **4.15** | 24.28 |
|  | Diabetic metabolic decompensation | 5 | 507 | 536 | 11821781 | 3.39 | **1.83** | 4.38 | 10.51 | **4.34** | 25.46 |
|  | Fournier’s gangrene | 4 | 508 | 882 | 11821435 | 3.06 | **1.30** | 4.14 | 8.36 | **3.12** | 22.41 |
|  | Gangrene | 4 | 508 | 1280 | 11821037 | 3.02 | **1.25** | 4.10 | 8.10 | **3.02** | 21.70 |
|  | Diabetes mellitus inadequate control | 3 | 509 | 4353 | 11817964 | 2.35 | **0.28** | 3.55 | 5.08 | **1.63** | 15.82 |
|  | Drug-induced liver injury | 3 | 509 | 9760 | 11812557 | 1.92 | -0.15 | 3.13 | 3.79 | **1.22** | 11.80 |
|  | Anal abscess | 3 | 509 | 2711 | 11819606 | 2.50 | **0.43** | 3.71 | 5.67 | **1.82** | 17.64 |

*In Table S3, the red bold text denotes significant signals. PTs: preferred terms; IC: Information Component; IC_025_: the lower end of the 95% confidence interval of IC; IC_975_: the upper end of the 95% confidence interval of IC; ROR: Reporting Odds Ratio; ROR_025_: the lower end of the 95% confidence interval of ROR; ROR_975_: the upper end of the 95% confidence interval of ROR; IC_025_ greater than 0 was deemed a signal, ROR_025_ over 1 with at least 3 cases was a signal.

# Table S4. The IC_025_ value of all SGLT2i-related important medical events in monotherapy-based on other antidiabetic drugs as "non-case".*

| SOC | PT | Records | Canagliflozin | Empagliflozin | Dapagliflozin | Ertugliflozin |
| --- | --- | --- | --- | --- | --- | --- |
| Metabolism and nutrition disorders | Diabetic ketoacidosis | 5716 | **0.68** | **0.94** | **0.96** | -1.28 |
|  | Ketoacidosis | 1468 | **0.32** | **1.03** | **0.87** | -2.15 |
|  | Euglycaemic diabetic ketoacidosis | 805 | -1.31 | **1.52** | **0.96** | -1.41 |
|  | Diabetic ketosis | 43 | - | **0.24** | **1.6** | - |
|  | Diabetic metabolic decompensation | 14 | - | -4.45 | -3.56 | **0.42** |
| Renal and urinary disorders | Acute kidney injury | 2686 | **0.16** | -2.13 | -2.07 | -3.35 |
|  | Renal failure | 527 | **1.39** | -1.64 | -1.93 | - |
|  | Nephrolithiasis | 108 | **0.47** | -0.17 | -0.62 | - |
|  | Chronic kidney disease | 89 | **0.01** | -1.35 | -2.15 | - |
|  | Renal injury | 56 | **1.29** | -3.6 | - | - |
|  | Urinary retention | 35 | -0.81 | -0.93 | **0.16** | - |
|  | Nephritis | 25 | - | -2.41 | **1.82** | - |
|  | Cystitis haemorrhagic | 21 | -2.21 | - | **1.5** | - |
|  | Azotaemia | 19 | **0.44** | - | - | - |
| Infections and infestations | Cellulitis | 912 | **1.78** | -2.09 | -2.04 | - |
|  | Fournier’s gangrene | 667 | -0.08 | **1.63** | **0** | -1.55 |
|  | Gangrene | 321 | **1.54** | -0.92 | -1.55 | -0.64 |
|  | Osteomyelitis | 228 | **1.76** | -2.83 | -5.01 | - |
|  | Necrotising fasciitis | 148 | -0.8 | **1.1** | **0.79** | - |
|  | Pyelonephritis | 76 | **0.38** | -0.09 | **0** | - |
|  | Urosepsis | 71 | -0.11 | **0.02** | **0.81** | - |
|  | Sepsis | 64 | -0.56 | -0.25 | **0.96** | - |
|  | Arthritis bacterial | 56 | **1.33** | -2.4 | - | - |
|  | Anal abscess | 39 | -1.53 | **0.79** | -0.57 | **0.24** |
|  | Kidney infection | 34 | -0.61 | **0.62** | -1.42 | - |
|  | Osteomyelitis acute | 24 | **1.25** | - | - | - |
|  | Cellulitis of male external genital organ | 23 | -0.95 | **0.11** | -0.04 | - |
|  | Pyelonephritis acute | 22 | -0.55 | -1.69 | **0.36** | - |
|  | Epididymitis | 19 | - | -0.6 | **1.12** | - |
|  | Cellulitis gangrenous | 21 | **0.93** | - | - | - |
|  | Appendicitis | 20 | -1.82 | -0.86 | **0.32** | - |
|  | Perineal abscess | 14 | - | **0.86** | - | - |
|  | Infected skin ulcer | 14 | **0.08** | -1.09 | - | - |
|  | Urinary tract infection fungal | 12 | **0.08** | - | - | - |
|  | Necrotising soft tissue infection | 11 | - | **0.69** | - | - |
|  | Gas gangrene | 12 | **0.8** | - | - | - |
|  | Septic shock | 10 | -1.94 | **0.12** | - | - |
|  | Perineal infection | 9 | - | **0.64** | - | - |
|  | Abdominal infection | 8 | **0.04** | - | - | - |
|  | Vulval abscess | 10 | - | **0.43** | - | - |
|  | Escherichia bacteraemia | 8 | - | - | **0.82** | - |
|  | Emphysematous pyelonephritis | 6 | - | **0.3** | - | - |
|  | Enteritis infectious | 7 | **0.2** | - | - | - |
| Nervous system disorders | Cerebrovascular accident | 5 | **0.63** | **0.13** | -0.61 | - |
|  | Cerebral infarction | 440 | **0.07** | **0.39** | **1.24** | - |
|  | Altered state of consciousness | 277 | -5.09 | **0.56** | -0.87 | - |
|  | Syncope | 84 | -1.44 | -2.75 | **0.83** | - |
|  | Transient ischaemic attack | 35 | -0.99 | -0.61 | **0.46** | - |
|  | Brain stem infarction | 32 | -2.31 | -2.41 | **0.87** | - |
|  | Lacunar infarction | 19 | -1.63 | -0.21 | **0.11** | - |
|  | Thrombotic cerebral infarction | 16 | - | -0.85 | **0.66** | - |
|  | Cerebellar infarction | 12 | - | **0.12** | -1.12 | - |
|  | Diabetic hyperosmolar coma | 11 | - | - | **0.43** | - |
|  | Haemorrhagic transformation stroke | 8 | - | - | **0.01** | - |
| Cardiac disorders | Myocardial infarction | 3 | **0.48** | -0.06 | -0.3 | - |
|  | Cardiac failure | 323 | -1.61 | **0.08** | -0.63 | - |
|  | Angina unstable | 152 | -2.83 | -1.91 | **0.19** | - |
|  | Coronary artery occlusion | 31 | - | -0.6 | **0.06** | - |
| Neoplasms benign, malignant and unspecified | Bladder cancer | 15 | -1.95 | -1.1 | **0.62** | - |
|  | Colon cancer | 164 | -0.84 | -1.12 | **0.03** | - |
|  | Breast cancer female | 41 | - | -0.06 | **0.89** | - |
|  | Renal cancer | 26 | -0.74 | -2.41 | **0.51** | - |
|  | Prostate cancer | 30 | -1.15 | -2.47 | **0.58** | - |
|  | Transitional cell carcinoma | 29 | -1.63 | - | **0.11** | - |
| Gastrointestinal disorders | Ileus | 10 | - | - | **0.42** | - |
| General disorders and administration site conditions | Death | 8 | -0.8 | -0.66 | **1.03** | - |
|  | Necrosis | 450 | -0.44 | - | **0.68** | - |
| Vascular disorders | Dry gangrene | 19 | **1.43** | - | - | - |
|  | Haemorrhage | 45 | -2.4 | -2.68 | **0.25** | - |
| Eye disorders | Retinal haemorrhage | 24 | **0.09** | - | - | - |
| Psychiatric disorders | Suicidal ideation | 8 | **0.08** | - | -1.44 | - |
|  | Near death experience | 13 | - | - | **0.34** | - |
| Musculoskeletal and connective tissue disorders | Neuropathic arthropathy | 5 | **0.44** | - | - | - |
| Blood and lymphatic system disorders | Disseminated intravascular coagulation | 11 | -2.13 | - | **0.58** | - |
| Social circumstances | Disability | 10 | -0.5 | **0.27** | -1.96 | - |

*In Table S4, the red bold text denotes significant signals. SOC: system organ class; PT: preferred term; IC: Information Component; IC_025_: the lower end of the 95% confidence interval of IC; IC_025_ greater than 0 was deemed a signal.





# Figure S2. Associations between the four common strong-signal PTs and different SGLT2i strategies quantified by IC values*

*In Fig.S2, PT: preferred term; IC: information component; Cana: canagliflozin; Empa: empagliflozin; Dapa: dapagliflozin; Ertu: ertugliflozin; Cana+Met: canagliflozin+metformin; Empa+Met: empagliflozin+metformin; Dapa+Met: dapagliflozin+metformin; Ertu+Met: ertugliflozin+metformin; Empa+Lina: empagliflozin+linagliptin; IC_025_ greater than 0 was deemed a signal.


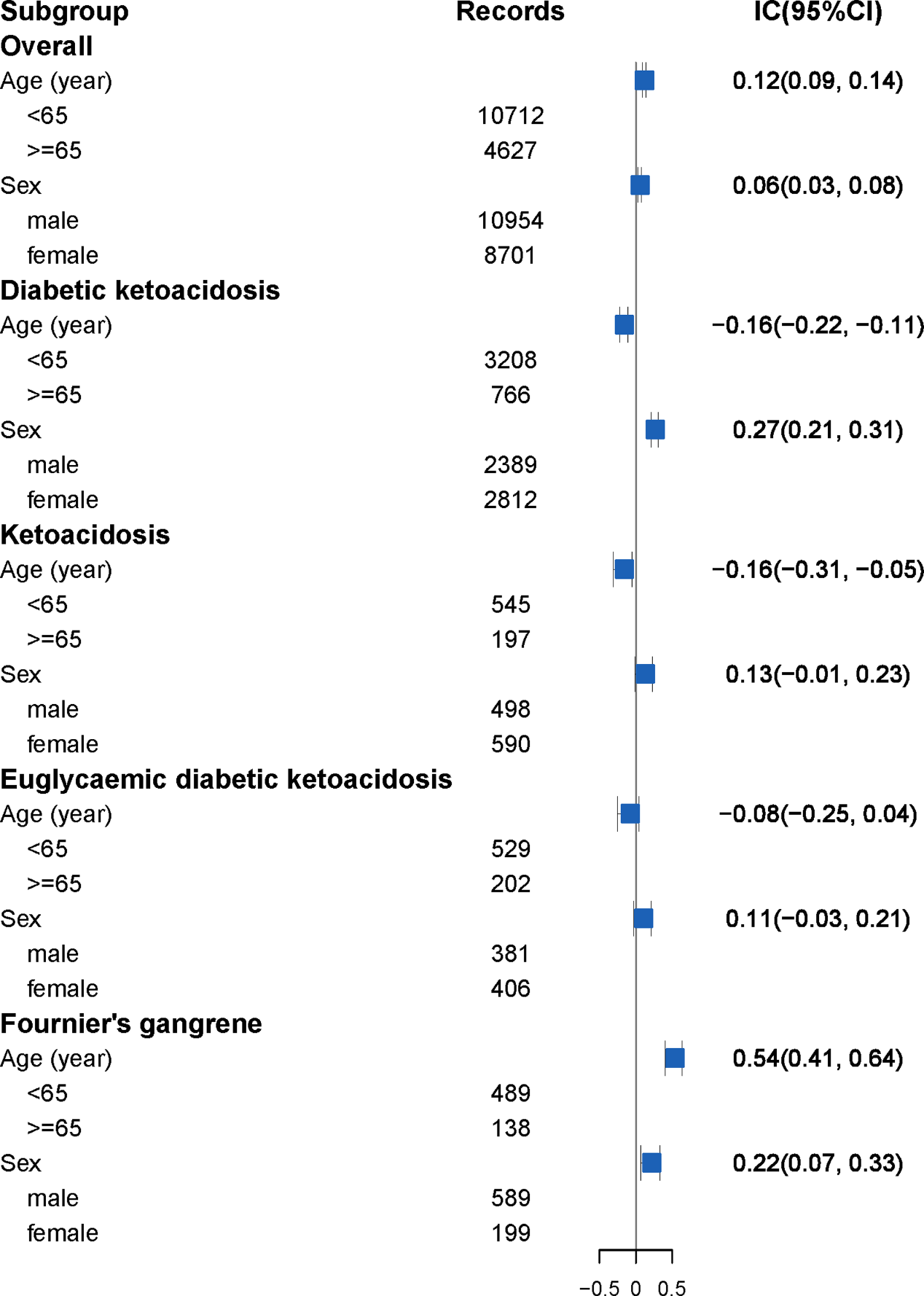


# Figure S3. Comparisons of SGLT2is-related IMEs (total and common) between age and gender*

*In Fig.S3, IC: information component; IC_025_ greater than 0 was deemed a signal.
